# Rapid 3D Immunolabeling and Light Sheet Microscopy for Quantitative Analysis of Intact Tissues

**DOI:** 10.34133/csbj.0121

**Published:** 2026-05-21

**Authors:** Junyu Chen, Zhangfan Ding, Lincoln Biswas, Jessica De Angelis, Alexandros Chatzis, Anjali P. Kusumbe

**Affiliations:** ^1^ Tissue and Tumor Microenvironments Lab, Cancer Discovery and Regenerative Medicine Program, Lee Kong Chian School of Medicine, Nanyang Technological University, Singapore.; ^2^ State Key Laboratory of Oral Diseases, National Clinical Research Center for Oral Diseases, Department of Prosthodontics, West China Hospital of Stomatology, Sichuan University, Chengdu, China.; ^3^ State Key Laboratory of Oral Diseases, National Center for Stomatology, National Clinical Research Center for Oral Diseases, Department of Head and Neck Oncology, West China Hospital of Stomatology, Sichuan University, Chengdu, China.; ^4^ Multidisciplinary Institute of Ageing (MIA-Portugal), Coimbra, Portugal.

## Abstract

Whole-organ 3-dimensional (3D) imaging of intact tissues provides high-resolution cellular and molecular insights into tissue and tumor microenvironments. However, immunolabeling and tissue clearing methods remain complex and time-consuming and often rely on toxic reagents and prolonged processing times. Here, we introduce a rapid 3D immunolabeling and light sheet microscopy platform for quantitative analysis of intact murine organs and human tissues within 2 to 2.5 d. This streamlined workflow integrates antigen retrieval, permeabilization, collagenase-based digestion, immunolabeling, dehydration, and tissue clearing into a single optimized pipeline for fast and reproducible processing of intact tissues. Notably, this ultrafast 3D imaging method is optimized for exogenous fluorescence labeling, overcoming limitations associated with endogenous fluorescence in conventional tissue clearing approaches. It enables robust quantitative analysis and detection of rare cell populations, including round α-smooth muscle actin (α-SMA)-positive cells in the thymus, while preserving overall tissue integrity and maintaining compatibility with downstream histology. Using this rapid whole-tissue imaging platform, we mapped lymphatic vessel networks across multiple organs and age groups, revealing age-associated expansion in specific endocrine tissues but not in other organs. Overall, this method provides a rapid, reproducible, and versatile approach for whole-organ and intact tissue imaging, enabling comprehensive mapping of complex tissue architectures and rare cells, and advancing quantitative light sheet-based tissue analysis and disease research.

## Introduction

Advanced 3-dimensional (3D) imaging approaches provide critical insights into the complex biological processes that underlie organ development, regeneration, repair, and pathology (1–8). To further advance our understanding of these intricate processes, it is essential to establish accurate cellular and molecular maps of tissues across diverse physiological and pathological states, including healthy, aged, diseased, and malignant conditions (9–21).

3D interrogation of intact biological specimens preserves tissue architecture and provides essential structural and spatial information, facilitating the exploration of cell–cell interactions and microenvironmental cues within their native contexts (9,22–28). Such analysis is especially critical for decoding the organization of complex systems, such as the nervous and vascular networks, which span entire organs and are often obscured by traditional thin-section histology (29–33). In addition, intravital imaging enables real-time visualization of dynamic processes in living tissues and organs, providing critical insights into vascular, neural, and microenvironmental interactions in their native context (34–37). However, the acquisition of these high-content datasets remains challenging, typically requiring extensive sample preparation, long processing times, large data volumes, and sophisticated analytical tools for data interpretation. These technical hurdles have been partially addressed by recent advances in tissue clearing, light sheet fluorescence microscopy (LSFM), and computational data processing (12,22,38–46).

Despite these technological gains, current protocols for immunostaining and whole-organ clearing represent time-consuming, labor-intensive, expensive, and organ-specific (47–53). The long processing time, often ranging from one to several weeks, remains a major barrier to applying whole-organ imaging to study biological complexity and cellular interactions. Other disadvantages include the requirement for transgenic mice expressing fluorescent reporters and the use of toxic or corrosive chemicals that may be detrimental to either the microscope or the researcher’s well-being (11,54,55). These limitations continue to impede the widespread adoption of whole-organ imaging in routine biological and translational research.

Therefore, it is essential to develop a rapid method for whole organs and large tissue multicolor immunostaining coupled with a technique that is applicable across diverse tissues while preserving endogenous fluorescence. In this study, we devised a robust and ultrafast 3D imaging method that enables immunostaining and clearing of multiple whole organs and large tissues in a record time of 2.5 d. The method also addresses several limitations of existing approaches by avoiding highly toxic organic solvents used in conventional clearing protocols and by enabling multicolor imaging with exogenous fluorescent labeling in both mouse and human tissues.

## Materials and Methods

### Mice

Organs and endocrine glands were collected from adult wild-type C57BL/6J mice (8 to 15 weeks old, both sexes), originally obtained from the Jackson Laboratory and subsequently maintained in-house. To assess age-related changes in lymphatic vessels, young mice aged 8 to 10 weeks and aged mice aged 56 to 70 weeks were analyzed. For experiments involving endogenous fluorescence, *Kdr^tm2.1Jrt^/J* mice (stock no.: 017006, Jackson Laboratory) (56) and *ROSA26 td-Tomato* reporter mice (stock no.: 007914, Jackson Laboratory) (57) were used. To genetically label vascular structures, *Cdh5(PAC)-CreERT2* transgenic mice were crossed with *ROSA26 td-Tomato* reporter lines. Further, *GFAP-GFP* reporter mice (stock no.: 010835, Jackson Laboratory) were used to assess fluorescence preservation across imaging depths following tissue clearing. All animals were maintained under standard conditions at the University of Oxford. Animal experiments were conducted in compliance with the UK Home Office regulations under the Animals (Scientific Procedures) Act 1986. All procedures were approved by the local Animal Welfare and Ethical Review Board and authorized by the UK Government Home Office (Animals Scientific Procedures Group).

### Tamoxifen treatment

For oral gavage administration, tamoxifen (Sigma, T5648) was initially dissolved in 100% ethanol and subsequently diluted in corn oil to a final concentration of 5 mg/ml. In *Cdh5(PAC)-CreERT2; ROSA26-td-Tomato* transgenic mice, Cre recombinase activity was induced by oral tamoxifen administration at 50 mg/kg body weight once daily for 3 consecutive days. Mice were euthanized and tissues were collected for analysis 2 weeks after the final tamoxifen dose.

### Evans blue tracing

A direct lymphatic tracing method was used to visualize lymphatic vessels in mouse tissues. Evans Blue (Sigma-Aldrich, E2129), a vital dye that binds strongly to tissue proteins and is preferentially taken up by initial lymphatic vessels from the interstitial space, was injected subcutaneously into the inner thigh, the medial base of the tail, and the footpad. The formation of slight blebbing at the injection site indicated successful intradermal delivery. After administration, the dye was transported through lymphatic vessels and its accumulation in organs was examined 4 h post-injection.

### Tissue collection and preparation

Freshly dissected mouse tissues were briefly rinsed in phosphate-buffered saline (PBS; VWR, 437117K) and immediately immersed in freshly prepared ice-cold fixative containing 4% paraformaldehyde (PFA) (Sigma-Aldrich, P6148) and 0.05% glutaraldehyde (Sigma-Aldrich, 340855) for up to 3 h. The addition of glutaraldehyde facilitates rapid crosslinking and improves preservation of tissue morphology and cellular architecture. To minimize potential adverse effects of fixation reagents, the fixation duration was strictly limited to no more than 3 h. All fixation and washing steps were performed in 50-ml centrifuge tubes containing at least 30 ml of solution to ensure complete immersion. During dissection, adjacent muscle and adipose tissue were carefully removed to prevent interference with subsequent whole-organ imaging.

After fixation, samples were washed 3 times in PBS at room temperature (RT) on a shaker, with each wash lasting 5 min to ensure sufficient removal of residual fixative. Fixed tissues can be stored at 4 °C for up to 4 d before proceeding to bleaching or antigen retrieval and permeabilization. Although immersion fixation is adequate for most murine organs, transcardial perfusion with 4% PFA is recommended when more rapid and uniform fixation is required. As PFA and glutaraldehyde are hazardous and potentially toxic fixatives, all handling procedures were carried out with appropriate personal protective equipment and in compliance with established laboratory safety guidelines.

### Bleaching

For organs with high heme content, a bleaching step was incorporated to improve overall optical transparency. Tissues were first dehydrated through a graded methanol series (50%, 80%, and 100%), each step lasting 40 min, with the 100% methanol replaced twice at 20-min intervals to ensure efficient dehydration. All dehydration solutions were pre-cooled and maintained at 4 °C. The entire dehydration, bleaching, and subsequent rehydration procedures were performed in 50-ml centrifuge tubes containing at least 30 ml of solution to ensure full tissue immersion. Bleaching was conducted by incubating samples in 5% (v/v) hydrogen peroxide in methanol for up to 3 h at 4 °C, with samples protected from light to prevent reagent degradation and photodamage. Following bleaching, tissues were gradually rehydrated through the reverse methanol gradient and subsequently washed 3 times in PBS for 20 min each to remove residual solvents. As an alternative, isopropanol may be used for dehydration prior to bleaching due to its comparatively lower toxicity. However, methanol provides superior depigmentation and more effective delipidation and enhances membrane permeabilization, thereby facilitating improved antibody penetration in downstream immunolabeling steps. As methanol and hydrogen peroxide are hazardous chemicals with toxic and oxidative properties, all procedures involving these reagents were conducted using appropriate personal protective equipment and in strict accordance with institutional laboratory safety guidelines.

### Antigen retrieval, permeabilization, and collagenase digestion

Following fixation, tissues underwent antigen retrieval and permeabilization in an ice-cold buffer composed of 25% urea (VWR, 28876.367), 15% glycerol (VWR, 24388.260), and 7% Triton X-100 (Sigma-Aldrich, T8787) diluted in ddH_2_O. Samples were incubated at 4 °C for 6 to 12 h depending on tissue size and density. For small murine organs such as the adrenal gland, 6 h was sufficient, whereas larger organs, including the heart and brain, required extended incubation up to 12 h to ensure adequate permeabilization. Antigen retrieval, permeabilization, and subsequent collagenase digestion were all performed in 50-ml centrifuge tubes containing at least 30 ml of solution to ensure complete immersion of the tissues. All reagents were freshly prepared before use, and any remaining solution was properly discarded according to laboratory safety regulations.

Subsequently, tissues were subjected to extracellular matrix digestion using freshly prepared 0.2% collagenase A (Merck, 10103578001) in PBS. Digestion was conducted at 37 °C for 30 min with continuous gentle shaking, and the incubation time was strictly limited to avoid excessive structural damage while maintaining sufficient permeability. After digestion, samples were washed twice for 5 min in PBS containing 2% fetal bovine serum (FBS) (Sigma-Aldrich, F7524) to remove residual enzyme before proceeding to the next steps.

### Immunolabeling

For immunolabeling, organs were placed in 5-ml tubes and incubated in a freshly prepared blocking solution consisting of 10% donkey serum (Abcam, ab7475), 10% dimethyl sulfoxide (DMSO), and 0.5% Triton X-100 in PBS at 37 °C for 20 min. The blocking and subsequent antibody incubation steps were performed in 5-ml tubes with sufficient solution volume to fully immerse the samples. After blocking, tissues were incubated either with Alexa Fluor-conjugated antibodies directly or with unconjugated primary antibodies diluted (1:500 for all antibodies listed in Table S1) in a staining buffer containing 2% donkey serum, 10% DMSO, and 0.5% Triton X-100 in PBS. Incubation was carried out for 14 to 16 h (or overnight) at 37 °C in a shaking water bath (Stuart, SBS40) at 70 rpm to ensure uniform antibody distribution and penetration. Following primary antibody incubation, samples were transferred to 15-ml tubes and washed in at least 10 ml of wash buffer (2% donkey serum and 0.5% Triton X-100 in PBS) for 3 h at 37 °C with continuous agitation at 70 rpm. The wash solution was replaced every 15 min during the first hour and every 30 min for the remaining 2 h to facilitate efficient removal of unbound antibodies. For samples stained with unconjugated primary antibodies, secondary antibody incubation was subsequently performed for 6 to 8 h at 37 °C under the same shaking conditions using staining buffer of identical composition. Nuclear counterstaining was performed using 4′,6-diamidino-2-phenylindole (DAPI), which was added during the secondary antibody incubation step. After secondary incubation, samples were washed again under the same conditions before proceeding to the next steps.

All secondary antibodies were used as follows: donkey anti-rat immunoglobulin G (IgG) Alexa Fluor 594 (A21209, Thermo Fisher Scientific), donkey anti-goat IgG Alexa Fluor 488 (A11055, Thermo Fisher Scientific), donkey anti-goat IgG Alexa Fluor 647 (A21447, Thermo Fisher Scientific), donkey anti-goat IgG Alexa Fluor 546 (A-11056, Thermo Fisher Scientific), Alexa Fluor 488 streptavidin conjugate (S11223, Thermo Fisher Scientific), Alexa Fluor 546 streptavidin conjugate (S11225, Thermo Fisher Scientific), donkey anti-rabbit IgG Alexa Fluor 488 (A21206, Thermo Fisher Scientific), and donkey anti-rabbit IgG Alexa Fluor 647 (A31573, Thermo Fisher Scientific).

### Dehydration and tissue clearing

After immunostaining, tissues were gradually dehydrated using a stepwise isopropanol series (30%, 50%, and 80%, v/v), with each step lasting 30 min at RT under gentle rotation to ensure uniform solvent exchange. Samples were then transferred to 100% isopropanol for 1 h, during which the solvent was replaced twice to promote complete dehydration. As isopropanol is a flammable solvent, it was handled in accordance with standard laboratory safety regulations. All dehydration and subsequent clearing steps were conducted in 50-ml centrifuge tubes containing sufficient solution to fully submerge the organs. Following dehydration, residual isopropanol was removed, and samples were rinsed twice in ethyl cinnamate (ECi; Sigma-Aldrich, 112372) for 5 min each to facilitate solvent transition. Clearing was performed using a mixture of 80% ECi and 20% poly(ethylene glycol) methyl ether methacrylate [PEGM; Sigma-Aldrich, 447943; average Mn ~300 g/mol; chemical structure: CH_₂_=C(CH_3_)COO(CH_2_CH_2_O)_n_CH_3_; CAS: 26915-72-0] with continuous gentle rotation at RT for at least 30 min, or until adequate transparency was achieved. ECi and PEGM are considered less hazardous than conventional organic clearing solvents; however, both are chemical reagents that require handling under appropriate ventilation and standard laboratory safety precautions.

### CUBIC method

CUBIC reagents were prepared as in a previous study (47), and they are cooled to RT. Fixed samples were treated with reagent 1 for 3 to 6 d, washed with PBS, immersed in 20% (w/v) sucrose in PBS, and frozen in O.C.T. compound at −80 °C overnight. The frozen sample was then thawed, washed with PBS, and subjected to immunostaining with the primary antibodies in 750 ml of 0.1% (v/v) Triton X-100, 0.5% (w/v) bovine serum albumin, and 0.01% (w/v) sodium azide in PBS for 3 d at 37 °C with rotation. The stained samples were then washed with 0.1% (v/v) Triton X-100 in PBS several times at 37 °C with rotation and then stained with the secondary antibodies in 0.1% (v/v) Triton X-100, 0.1% (w/v) bovine serum albumin, and 0.01% (w/v) sodium azide in PBS for 3 d at 37 °C with rotation. The samples were then washed with 0.1% (v/v) Triton X-100 in PBS several times, immersed in 20% (w/v) sucrose in PBS, degassed, and immersed in reagent 2 for 24 to 36 h.

### ScaleS method

After deep anesthesia with pentobarbital, the entire vasculature of a mouse was transcardially perfused with ice-cold Hanks’ balanced salt solution (HBSS) solution containing a fluorochrome to label blood vessels. After perfusion with 4% PFA, the sample was resected and subjected to postfixation in 4% PFA at 4 °C for 10 h. The ScaleS solutions were prepared according to the previous protocol (48). Fixed mouse samples were incubated in a solution containing antibodies and propidium iodide (PI) at 37 °C for 6 h and then cleared by ScaleS clearing media.

### ECi-based method

Mice were transcardially perfused with 15 ml of cold PBS and 5 mM EDTA and perfusion fixed with 15 ml of cold 4% PFA/PBS (pH 7.4). After perfusion, organs were removed and postfixed in cold 4% PFA/PBS at 4 °C. After perfusion and postfixation, samples were dehydrated with ethanol. After dehydration, the samples were transferred to ECi and incubated while gently shaking at RT until they became transparent.

### Human samples

Formalin-fixed paraffin-embedded (FFPE) blocks of normal human testis, ovary, and thyroid gland were obtained from AMS Biotechnology (Oxford, UK). Human specimens supplied by AMS Biotechnology (Europe) Limited were procured in accordance with the applicable laws and regulations of the country of origin. All tissues were collected from consented donors at major research or clinical centers under protocols approved by local Ethics Committee/Institutional Review Board (EC/IRB) committees. Samples from donors aged 18 to 20 years and 70 to 80 years were selected to represent the young and aged groups, respectively. Histological examination was conducted to verify that the tissues were healthy and free of pathological alterations. Additional information regarding the human tissue samples is provided in Table S2.

### Imaging setup, image acquisition, and analysis

Cleared samples were imaged using a Miltenyi-LaVision Biotech UltraMicroscope II controlled by LaVision BioTech ImSpector software [MXDA-based Aqueous Clearing System (MACS), Miltenyi Biotec]. The system was equipped with a 2× objective for the zoom body with a manual zoom range of 0.63× to 6.3×. The objective was fitted with a dipping cap (5.7 mm) containing correction optics for the Olympus MVPLAPO 2× lens. The microscope was configured with laser lines at 405 to 100 nm, 488 to 85 nm, 561 to 100 nm, 639 to 70 nm, and 785 to 75 nm, and images were captured using a Neo sCMOS camera (Andor). For image acquisition, cleared samples were attached to the sample holder adapter using a small drop of Superglue (No Nonsense, UK). After securing the sample to the holder, it was gently immersed in ECi within a quartz glass cuvette and illuminated with light sheets (30 to 90 μm, depending on organ size) at excitation wavelengths of 488, 561, 640, and 785 nm. The resulting TIFF image stacks were converted using Imaris File Converter (version 9.6.1, Bitplane) and subsequently analyzed with Imaris software (version 9.6.0, Bitplane).

Confocal immunofluorescence images of whole organs or selected regions were acquired using a Zeiss Laser Scanning Microscope (LSM) 880 equipped with 7 laser lines (405, 453, 488, 514, 561, 594, and 633 nm), an Axio Examiner upright stand, and a Colibri7 epifluorescence light source with light-emitting diode (LED) illumination. A 10× Plan-Apochromat objective [numerical aperture (NA) 0.45, working distance (WD) = 2.0, M27, dry] was used to capture large areas using the tile scan function. Tile numbers were determined according to organ size, and images were acquired with 10% overlap to allow stitching in ZEN Black software (version 3.1, Zeiss). Samples were imaged across the *z* axis, and image stacks were reconstructed in Imaris to generate maximum intensity projections (MIPs). To visualize organ boundaries, autofluorescence detected in the 405-nm channel was converted to grayscale and overlaid at 30% opacity with the corresponding TIFF images generated from Imaris. Imaris, Adobe Photoshop, and Adobe Illustrator were used for image processing, analysis, and figure preparation.

### 3D surface rendering

3D surface rendering was performed using the Surface module in Imaris to visualize structural components within the organ. Initially, the whole organ or a defined region of interest (ROI) was selected for analysis. Surface smoothness was then adjusted using the Smooth parameter, and the background subtraction threshold was manually determined based on the preview of each image.

### Quantifications of imaging data

To quantify vascular density in whole organs, analyses were performed using the Imaris Surface Analysis XTensions tool. Total tissue volume was determined by applying the 3D crop function followed by the Volume statistics module in Imaris. Lymphatic structures labeled by a single-channel marker were reconstructed using the 3D surface rendering function, and vessel volume was measured using the Surface statistics tool. Lymphatic vessel density was calculated by dividing the measured vessel volume by the total tissue volume.

Quantification of Prox1^+^ cell numbers was performed using Imaris software (version 9.2.1) based on single-cell resolution 3D images. Automated segmentation and analysis were carried out using the nuclei detection and membrane detection functions within the Imaris Cell module. In the Cell Creation wizard, the Prox1 channel was selected as the source channel for membrane-based detection, while the DAPI channel was used for nuclei-based detection. Detected nuclei served as seed points for the membrane segmentation algorithm, enabling identification of cell boundaries. The number of Prox1^+^ cells per tissue volume (mm^−3^) was calculated by dividing the total number of segmented cells by the corresponding tissue volume.

Quantification of pulmonary alveolar size and renal corpuscle size was performed using ImageJ software (version 1.52). Briefly, regions corresponding to pulmonary alveoli and renal corpuscles were manually selected, and their areas (mm^2^) were measured to represent alveolar and renal corpuscle size.

### Quantitative evaluation of method performance

#### Linear shrinkage ratio and volume shrinkage ratio

Tissue deformation induced by the clearing process was evaluated using bright-field images acquired before and after processing. For each organ, the maximal anatomical length was measured using ImageJ, and the linear shrinkage ratio was calculated as the ratio between post-clearing and pre-clearing lengths. Assuming approximately isotropic deformation, the volume shrinkage ratio was estimated as the cubic power of the linear shrinkage ratio. For selected samples, projected area measurements were additionally recorded to confirm consistency between linear and volumetric estimations.

#### Signal-to-background ratio

To evaluate signal homogeneity across imaging depth, the signal-to-background ratio (SBR) was calculated from *z*-projection images. For each selected region, signal areas were segmented using a fixed threshold in ImageJ, and the mean intensity of the positive region was extracted. Background intensity was determined by averaging pixel values from multiple signal-free regions within the same projection. SBR was computed as:

$$\mathrm{SBR}=I_{\text{signal}}/I_{\text{background}}$$ (1)

where *I_signal_* and *I_background_* represent the mean intensities of the segmented signal and background regions, respectively.

#### Fluorescence preservation and normalized mean fluorescence

To determine fluorescence stability during clearing and storage, fluorescence preservation was quantified using normalized mean fluorescence values. For each sample and time point, MIP images were generated from defined cortical regions. Signal and background were separated using threshold-based segmentation. The corrected mean fluorescence intensity (M) was calculated as:

$$M=\frac{\sum I_{\text{signal}}}{N_{\text{signal}}}-\frac{\sum I_{\text{backgroud}}}{N_{\text{backgroud}}}$$ (2)

where ∑𝐼 denotes total pixel intensity and 𝑁 denotes the number of pixels within signal or background regions.

Fluorescence preservation immediately after clearing was expressed as *M*_0_/*M*_b_, where *M*_b_ represents mean fluorescence before clearing and *M*_0_ represents mean fluorescence at day 0 post-clearing. Long-term fluorescence stability was expressed as *M*_7_/*M*_0_ (or *M_i_*/*M*_0_ for general time point *i*), reflecting the relative retention of fluorescence during storage.

#### Voxel size and minimum detectable structure size

Voxel size was determined from acquisition metadata for each dataset, including the XY pixel size and Z-step size. For light sheet imaging, the XY pixel size was determined by the effective magnification and camera pixel size, whereas the Z-step size corresponded to the optical section interval set during acquisition. For representative datasets, the Z-step size was further verified by dividing the total imaging depth by the number of acquired optical sections. The minimum detectable structure size was empirically defined as the smallest antibody-positive tubular or cellular structure that could be clearly resolved and consistently segmented across at least 2 consecutive optical sections. Measurements were performed using Imaris software and ImageJ software on representative ROIs. Only structures with signal intensity above the segmentation threshold and continuous spatial representation across adjacent sections were included. These parameters were used to define the spatial scale of quantitative analyses and to support the reliability of vessel density and cell number measurements.

#### Depth-dependent fluorescence assessment

To assess depth-dependent fluorescence preservation, cleared mouse brain tissues were imaged by light sheet microscopy across the full imaging depth. Representative optical sections were extracted from superficial to deep regions, covering depths from 100 μm to approximately 7,000 μm. GFAP-GFP and DAPI signals were visually evaluated across these depths to assess fluorescence detectability and depth-dependent signal attenuation under the applied clearing and imaging conditions.

### Histology

Mice were sacrificed, and organs (liver, kidney, spleen, and heart) were collected. One set of organs was fixed in 10% formalin in PBS, while another set was fixed in 10% formalin and subsequently processed according to the clearing protocol (graded isopropanol dehydration followed by ECi clearing). All tissues were embedded in paraffin and sectioned into 5-μm slices. After hematoxylin and eosin (H&E) staining, sections were imaged using bright-field microscopy (Olympus, Japan).

### Quantitative PCR

For analysis of *Prox1* expression in FFPE blocks of human endocrine tissues, 25-μm sections were prepared from the FFPE samples. Following deparaffinization, sections were immersed in xylene and then washed twice in 100% ethanol before being air-dried for 15 min. Total RNA was extracted using an RNA extraction kit (QIAGEN, 73504) according to the manufacturer’s instructions. The isolated RNA was immediately used for cDNA synthesis with the SuperScript IV First-Strand Synthesis System (Invitrogen, 18091200). Quantitative polymerase chain reaction (qPCR) was performed using TaqMan gene expression assays on an ABI PRISM 7900HT Sequence Detection System. FAM-labeled TaqMan probes were used together with TaqMan Gene Expression Master Mix (Applied Biosystems, 4369510). Relative gene expression levels were normalized to *Actb*.

### Statistical analysis

All statistical analyses were performed using GraphPad Prism software (version 9.1) and R software (version 4.5.1). All data are presented as mean ± SD. Data distribution was first assessed for normality using the Shapiro–Wilk test, with *P* > 0.05 considered indicative of a normal distribution. Homogeneity of variances between groups was evaluated using Levene’s test, with *P* > 0.05 indicating equal variances. When both assumptions were satisfied, comparisons between 2 groups were typically assessed using 2-tailed unpaired Student’s *t* tests, while comparisons among multiple groups were evaluated using one-way analysis of variance (ANOVA). *P* values were further adjusted for multiple testing using the false discovery rate (FDR) method. The statistical significance was declared when *P* < 0.05. ns: not significant; *P < 0.05; **P < 0.01; ****P < 0.001; ****P < 0.0001. No randomization or blinding was used, and no animals were excluded from analyses. Several independent experiments were performed to guarantee reproducibility of findings.

## Results

### An ultrafast 3D immunolabeling and clearing method

**Fig. 1.**
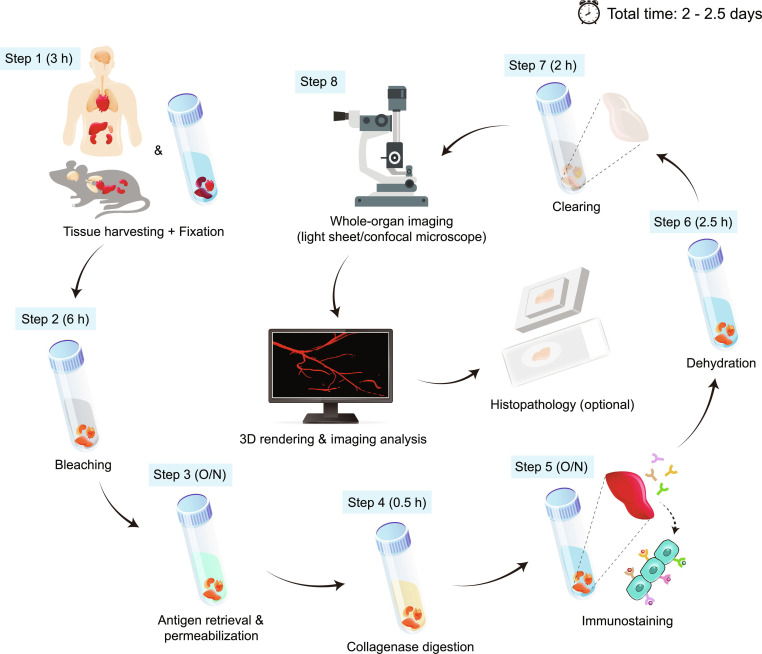
Whole-organ imaging using the simple ultrafast 3D imaging and clearing method. Schematic illustration of the ultrafast 3D imaging workflow for whole organs and large tissues. After tissue harvesting and fixation (step 1), samples undergo bleaching (step 2, optional), antigen retrieval and permeabilization (step 3), collagenase digestion (step 4), multicolor immunostaining (step 5), dehydration (step 6), and clearing (step 7). Cleared organs are imaged using a light sheet or confocal microscope (step 8), and Z-stacks are subsequently rendered and analyzed in 3D. After imaging, samples can also be processed for histopathology (optional). The time shown for each step represents the maximum duration required. The total processing time is approximately 2.5 d. O/N, overnight.

In this method, we developed a platform that enables multicolor immunolabeling while preserving optical cellular morphology, tissue architecture, and transparency across multiple murine organs and human tissues. Each of the 8 steps was optimized to minimize processing time (Fig. 1).

First, we combined PFA and glutaraldehyde for rapid fixation (step 1). Samples were bleached with hydrogen peroxide in methanol to eliminate heme and other persistent chromophores (step 2). Urea facilitated antigen retrieval, and Triton X-100 increased antibody penetration into tissues while preserving tissue architecture (step 3). Collagenase digestion of the extracellular matrix, using freshly prepared collagenase A and gentle shaking to ensure uniform and efficient treatment, markedly enhanced antibody penetration while preserving tissue architecture (step 4). To obtain optimal staining, only antibodies conjugated with small, bright fluorophores with high stability and good tissue penetrance were used (step 5). Triton X-100, a mild detergent, was included for blocking, staining, and washing the organs during this step. Incubations with antibodies and subsequent washes were performed at 37 °C to further the processing time. Dehydration is a crucial step that precedes tissue clearing and refractive index (RI) matching (step 6). Dehydration with isopropanol preserved all fluorophores and resulted in less tissue shrinkage than ethanol. ECi is considered less hazardous than many conventional organic solvents and has been utilized for tissue clearing. PEGM has also been used as a component of tissue clearing solutions. For this method, ECi (80%) and PEGM (20%) were combined. Compared with ECi alone, the combination of ECi and PEGM (RI: 1.534) resulted in faster and more efficient tissue clearing, achieving a higher degree of transparency (Fig. S1). This combination cleared all the tested organs and tissues within 2 h (step 7). ECi was used for RI matching for imaging (step 8). From organ collection to image acquisition, this protocol for multicolor antibody labeling and tissue clearing required up to 2.5 d with conjugated antibodies (Fig. 1). Finally, after this 3D imaging method, the clarified tissues could be sectioned for histopathology analysis. To enhance usability and reproducibility, detailed troubleshooting tips relevant to this method can be found in Table S3.

Overall, this method offers a streamlined and user-friendly workflow that markedly shortens whole-organ processing time from the conventional duration of one to several weeks to approximately 2.5 d. The protocol facilitates efficient antibody penetration and supports multicolor labeling with exogenous fluorophores, thereby enhancing the versatility of 3D structural visualization. Moreover, since clearing solutions are used during both sample preparation and prolonged immersion during imaging, the use of lower-hazard clearing reagents in this 3D imaging method further ensures a safer working environment for researchers.

### Enabling whole-organ 3D visualization of mouse and human tissues

**Fig. 2.**
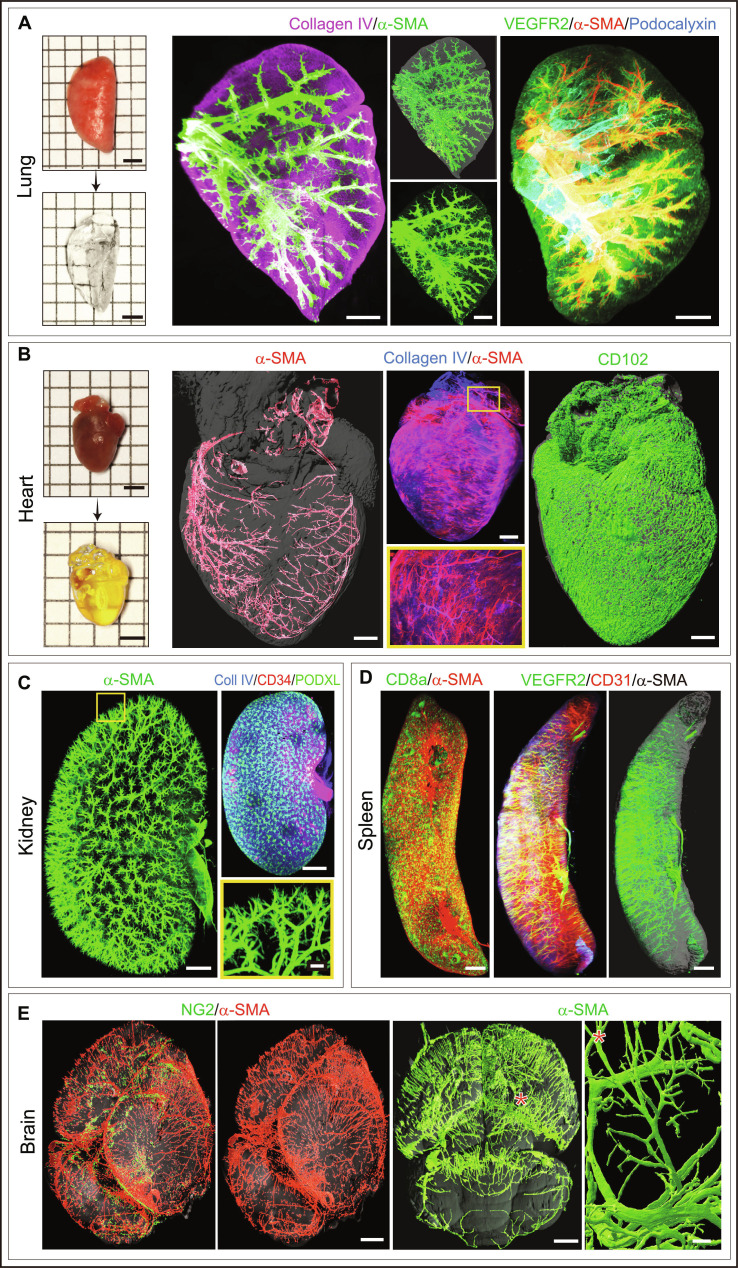
Light sheet imaging of whole cleared organs using this method. (A) Photos show mouse lung transparency before and after clearing. Light sheet images of cleared lung immunostained with collagen IV and α-SMA (middle) and VEGFR2, α-SMA, and Podocalyxin (right). (B) Photos show mouse heart transparency before and after clearing. Imaging of cleared heart stained as indicated, with inset showing higher magnification. (C) 3D imaging of cleared mouse kidney stained as indicated, with inset showing higher magnification. (D) 3D imaging of cleared spleen immunostained with CD8a and α-SMA (left) and VEGFR2, CD31, and α-SMA (right). (E) 3D imaging of cleared brain immunostained with NG2 and α-SMA. Inset (asterisk) shows higher magnification regions. Scale bars: 500 μm (A to D); 700 μm for whole-organ images and 200 μm for insets (B); 500 μm for whole-organ images and 50 μm for insets (C and E); 2 mm for photos in (A) and (B).

**Fig. 3.**
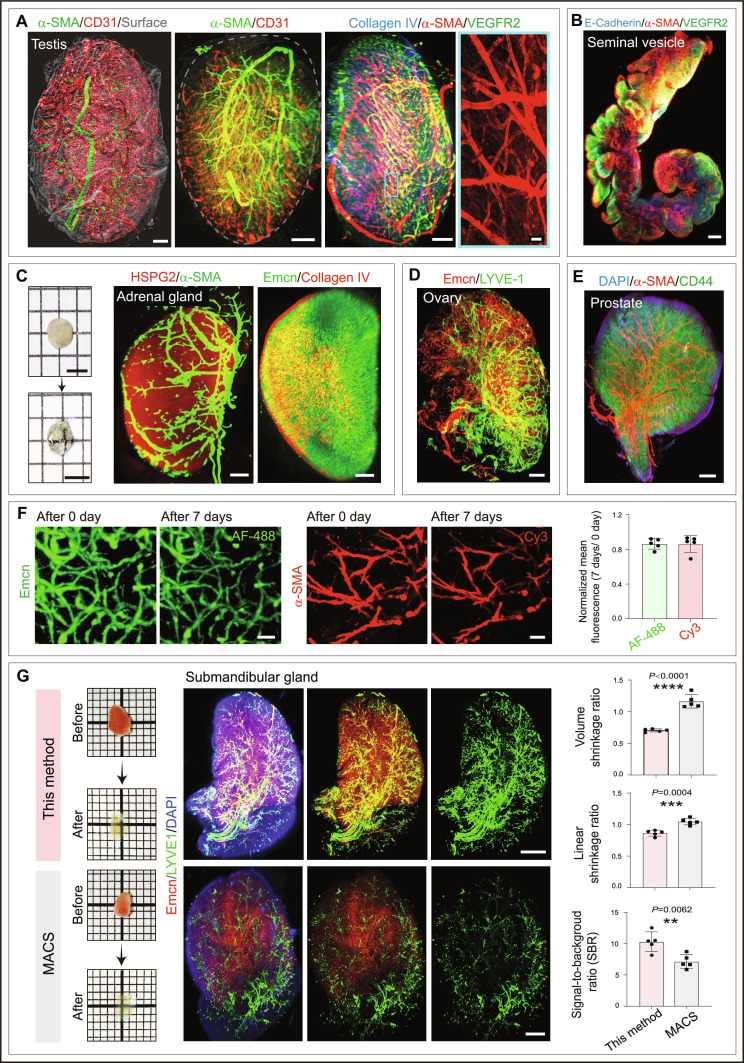
Light sheet imaging of whole cleared endocrine glands using this approach. (A) 3D images of cleared mouse testis immunostained with α-SMA and CD31 (left and middle) and collagen IV, α-SMA, and VEGFR2 (right). Inset shows higher magnification of a specific region. (B) 3D imaging of cleared mouse seminal vesicle stained with E-cadherin, α-SMA, and VEGFR2. (C) Photos show mouse adrenal gland transparency before and after clearing. Light sheet 3D imaging of cleared adrenal gland immunostained with HSPG2 and α-SMA (left) and Emcn and collagen IV (right). (D) Whole-organ imaging of cleared mouse ovary immunostained with Emcn and LYVE-1. (E) 3D image of cleared mouse prostate gland immunostained with α-SMA, CD44, and DAPI. (F) 3D images of cleared mouse brain samples stained with Emcn and α-SMA, imaged immediately after clearing and after 7 d of storage. Quantifications of normalized mean fluorescence (7 d/0 d) (*n* = 5). (G) Photos and 3D images of the mouse submandibular glands processed by this method and MACS method, respectively. Quantifications of volume shrinkage ratio, linear shrinkage ratio, and signal-to-background ratio (SBR) (*n* = 5). Scale bars: 400 μm (A, C, D, and G); 400 μm for whole-organ image and 50 μm for inset; 900 μm (B); 500 μm (E); 2 mm for photos in (C); and 50 μm (F). Data represent mean ± SD. *P* values were derived from 2-tailed unpaired *t* tests. **P < 0.01; ***P < 0.001; ****P < 0.0001.

We validated this method across multiple murine organs, including lung, heart, kidney, spleen, liver, brain, gut, tongue, thymus, prostate gland, seminal vesicle, and endocrine glands (Figs. 2 and 3A to E and Fig. S2). This approach enabled clear visualization of the 3D architecture of vascular and lymphatic networks throughout intact organs, as well as associated perivascular cells and pericytes. We also provide representative 3D imaging videos highlighting the spatial organization and cellular interactions within intact organs (Movies S1 to S5). Notably, a few organs, such as the heart, retained a slightly brownish yellow color even after clearing (Fig. 2B); however, this had minimal impact on light sheet penetration depth. We tested approximately 40 antibodies to test and validate this method across multiple organs and tissues. We selected antibodies targeting cellular and extracellular matrix molecules expressed across different organs. Markers defining vascular and tissue microenvironments, including endothelial cells, pericytes, mesenchymal cells, and immune cells, were used. Endothelial cell markers included Endomucin (Emcn), Endoglin (CD105), CD31 (also known as PECAM1), CD102 (ICAM2), VEGFR2, and Podocalyxin. α-Smooth muscle actin (α-SMA) staining was used for artery quantification, and pericytes were identified by NG2. Lymphatic vessels were analyzed using LYVE-1, Prox1, and Podoplanin. We provide detailed information for each of these antibodies and their working conditions (Table S1). With these cell surface markers, this method enabled 3D imaging of blood vessels and associated niches across several organs and endocrine glands (Figs. 2 and 3A to E, Fig. S2, and Movies S1 to S5). Further, the estimated voxel size and minimum detectable structure size of this method are provided in Table S4.

This method maintained fluorescence signals well over time. For both Alexa Fluor 488 and Cy3, the normalized mean fluorescence remained at above 80% of the day 0 level after 7 d of storage following clearing (Fig. 3F), indicating effective fluorescence retention. Using the mouse submandibular gland as a model tissue, we further compared our method with MACS, one of the fastest currently available clearing methods. Although this method induced slightly greater volume and linear shrinkage, it produced more continuous and better-defined staining signals with a higher SBR (Fig. 3G), allowing improved visualization of tissue microstructure and network features. In addition, compared with additional methods such as CUBIC, ScaleS, and an ECi-based protocol, this method offers a clear advantage in processing time while maintaining comparable organ transparency and imaging quality (Fig. S3).

**Fig. 4.**
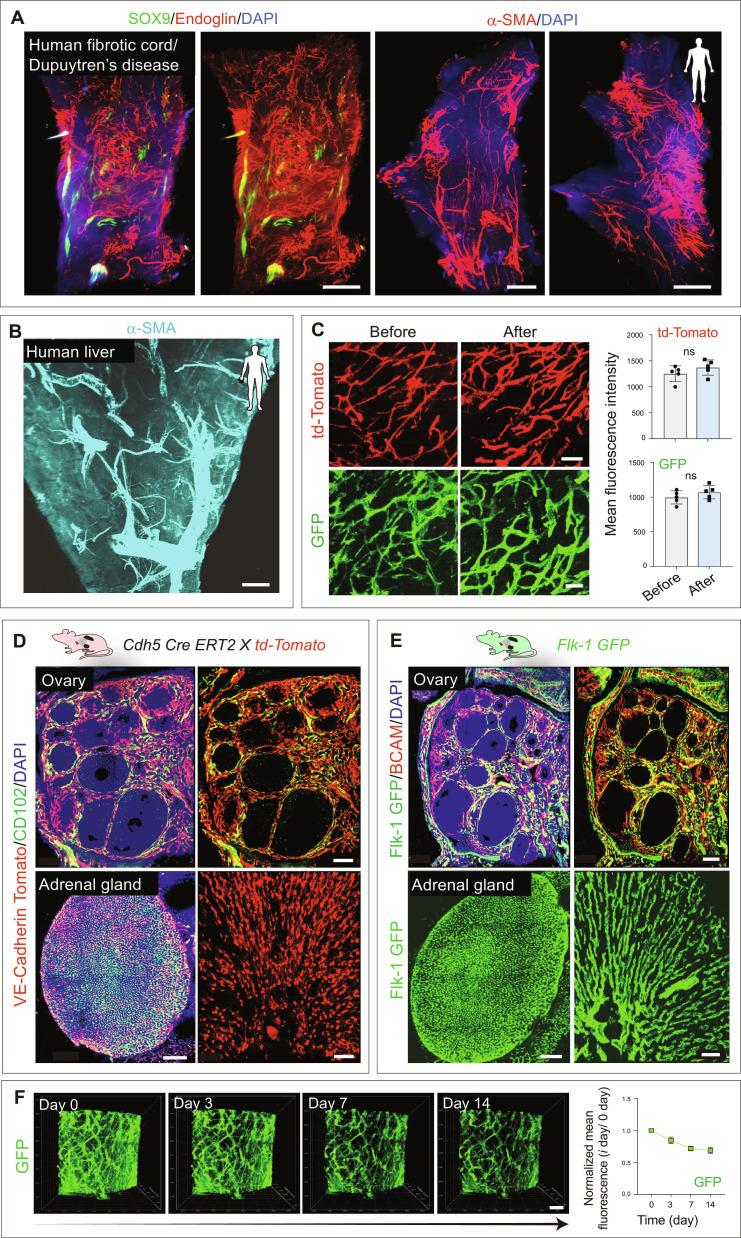
3D imaging of whole cleared endocrine glands and human tissues. (A) Light sheet image of Dupuytren’s disease cord tissue immunostained with SOX9 and Endoglin (left) and α-SMA (right). Nuclei: DAPI. (B) 3D imaging of cleared human liver with α-SMA immunostaining. (C) 3D confocal images of brain tissue before and after tissue clearing, obtained from *Cdh5(PAC)-CreERT2; ROSA26-td-Tomato* mice and *Flk1 GFP* reporter mice. (D) 3D images of cleared mouse ovary and adrenal gland stained with CD102 from *Cdh5(PAC)-CreERT2*; *ROSA26-td-Tomato* mutant mice acquired by confocal microscopy. Nuclei: DAPI. (E) 3D images of cleared ovary and adrenal gland immunostained with BCAM from *Flk1 GFP* reporter mice. Nuclei: DAPI. (F) 3D images of mouse brain tissue at 0, 3, 7, and 14 d after tissue clearing. Quantification of normalized mean fluorescence (*i* d/0 d) (*n* = 5). Scale bars: 400 μm (A and B); 50 μm (C and F); 150 μm for tile scan images and 50 μm for high-magnification insets (D and E). Data represent mean ± SD. *P* values were derived from 2-tailed unpaired *t* tests. ns, not significant.

In addition to the murine organs, this method permitted ultrafast immunostaining and clearing of human tissues (Fig. 4A and B). Using this approach, we mapped the hepatic arterial architecture (Fig. 4B) and also visualized vascular networks and key regulatory markers in human disease tissues, such as fibrotic cord tissues in Dupuytren’s disease (Fig. 4A). This approach offers a promising tool for histopathological analysis and potential diagnostic applications in human diseases.

### Preservation of tissue architecture and endogenous fluorescence

To determine the effect of this method on tissue morphology, we embedded the processed organs in paraffin and examined the samples, which had previously been imaged in 3D by conventional histological analysis. 2D histology showed that tissues processed by this method remained accessible to conventional H&E (Fig. S4). Tissue architecture remained intact, and structures such as pulmonary alveoli and renal corpuscles were clearly identifiable in tissues processed with this method, similar to control tissues processed by conventional paraffin embedding (Fig. S4). Using the mouse submandibular gland as a model, we strictly controlled digestion conditions by limiting collagenase A to 0.2% and the incubation time to within 30 min. Under these conditions, KLK1 staining showed that the ductal network of the salivary gland remained structurally intact after digestion (Fig. S5A), indicating that the treatment did not compromise duct integrity. In contrast, nondigested samples showed markedly weaker signals in deeper regions (Fig. S5A), limiting visualization of ductal structures. Further, the digestion process did not affect the quantitative assessment of vasculature in the mouse salivary glands (Fig. S5B). Thus, this approach enabled rapid, deep tissue immunolabeling and clearing of intact organs while maintaining tissue architecture.

Loss of endogenous fluorescence is a crucial concern for solvent-based tissue clearing medium and other steps during tissue processing. To assess the impact of clearing on endogenous fluorescence, either *Flk1 GFP* or *CDh5 Cre ERT2 X td-Tomato* mice were used. No significant differences in mean fluorescence intensity of td-Tomato and GFP were observed before and after clearing (Fig. 4C). Our analysis further revealed that mouse ovary and adrenal gland samples processed by this method preserved td-Tomato and GFP fluorescence (Fig. 4D and E). To evaluate long-term preservation of endogenous fluorescence, GFP signals were monitored after clearing. Normalized mean fluorescence declined during the first few days, stabilized after approximately 7 d, and remained detectable for up to 14 d (Fig. 4F), indicating good long-term preservation of endogenous fluorescence. To evaluate depth-dependent fluorescence preservation, cleared *GFAP-GFP* mouse brain tissues were imaged across the full imaging volume using light sheet microscopy. Both GFAP^+^ astrocyte and DAPI signals were preserved and detectable up to ~7 mm in depth (Fig. S5C). For larger heme-rich organs, bleaching with 5% H_2_O_2_ is required. Given that GFP is sensitive to hydrogen peroxide, anti-GFP staining can be used to avoid fluorescence quenching.

### This method enables region-specific cellular analysis within whole organs

**Fig. 5.**
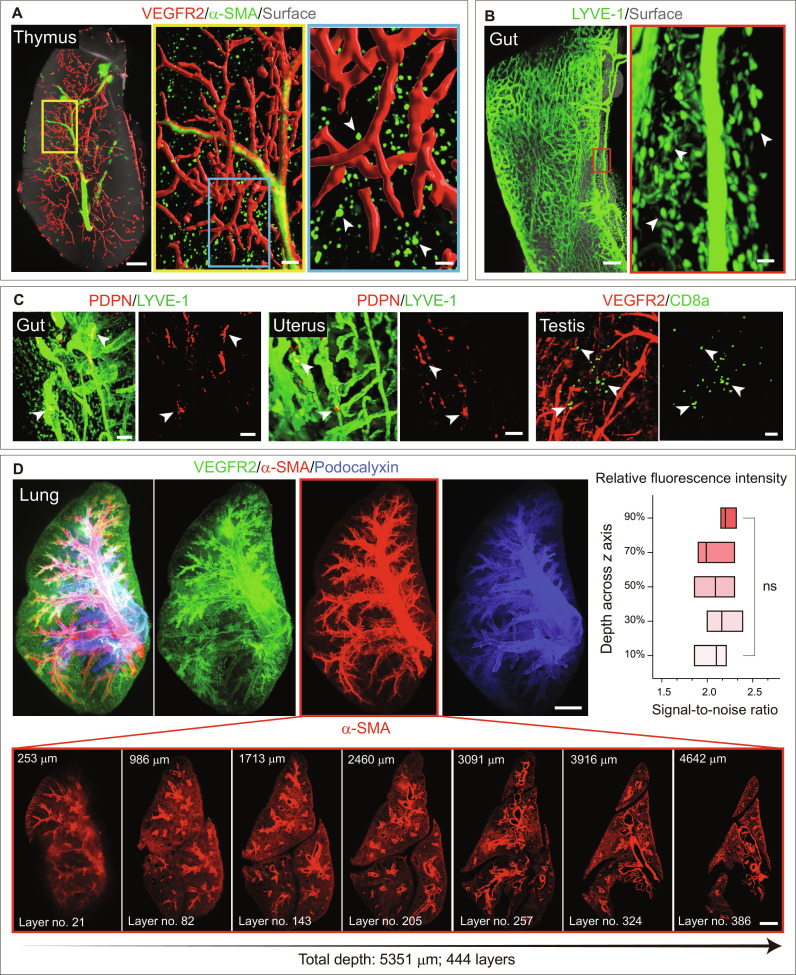
Single-cell resolution analysis and detection of rare cells in whole organs using this method. (A) Whole-organ imaging of cleared mouse thymus stained with VEGFR2 and α-SMA acquired on a light sheet microscope. Insets show higher magnification of specific regions. Arrowheads indicate α-SMA^+^ stromal cells. (B) Whole-organ imaging of cleared mouse gut stained with LYVE-1. Inset shows higher magnification of a specific region. Arrowheads indicate LYVE-1^+^ macrophages. (C) 3D images (left and middle) of cleared gut and uterus stained with Podoplanin (PDPN) and LYVE-1. Arrowheads indicate Podoplanin^+^ lymphatic endothelial cells. 3D images (right) of cleared testis stained with VEGFR2 and CD8a. Arrowheads indicate CD8a^+^ cells. (D) Whole-organ imaging (top) of cleared mouse lung stained with VEGFR2, α-SMA, and Podocalyxin acquired on a light sheet microscope. Representative section gallery views (bottom) of 444 longitudinal sections across the lung with a depth of 5,351 μm. Plots show quantification of relative fluorescence intensity at different depth positions (*n* = 4). Scale bars: 400 μm for whole-organ image, 150 μm for middle inset, and 50 μm for right inset (A); 300 μm for whole-organ image and 50 μm for inset (B); 50 μm (C); 500 μm (D). Data represent mean ± SD. *P* value derived from one-way ANOVA. ns, not significant.

Light sheet microscopy was used for volumetric imaging of murine organs and human tissues processed with this method, generating whole-organ and tissue-level overviews. We next sought to transition from global tissue visualization to region-specific analysis within organs. Computational surface rendering was subsequently applied to selected ROIs, enabling detailed visualization of cellular structures and specific cell populations within 3D tile scan datasets (Fig. 5A to C). These results indicate that the ultrafast method facilitates visualization of complex cellular networks, including vascular structures, as well as spatially localized individual cells within intact organs.

This analysis revealed a small subset of round α-SMA-positive cells within the thymic cortex. Notably, these cells were detected in addition to their usual periarteriolar location (Fig. 5A). Such round α-SMA-positive monocyte–macrophage subsets have been reported in the bone marrow, where these cells maintain primitive hematopoiesis (58). However, the presence of such a small subset of α-SMA-positive monocytes/macrophages has not been previously reported in the thymus. Moreover, this method enabled the clear visualization of LYVE-1^+^ and Podoplanin^+^ lymphatic endothelial cells (LECs) in the gut, Podoplanin^+^ LECs in the uterus, and CD8a^+^ cells in the testis (Fig. 5B and C). In addition, this approach allows for precise vascular dye tracing, as exemplified by functional lymphatic vessel tracing in the pancreas using Evans Blue (Fig. S6), further highlighting its robust imaging depth. The imaging performance of this method is associated with effective antibody penetration across intact tissues. Notably, in representative organs, immunolabeling signals remained uniformly distributed across the entire imaging depth (Fig. 5D and Fig. S7), indicating consistent antibody access and precise spatial preservation.

### This method enables quantitative imaging of whole organs

**Fig. 6.**
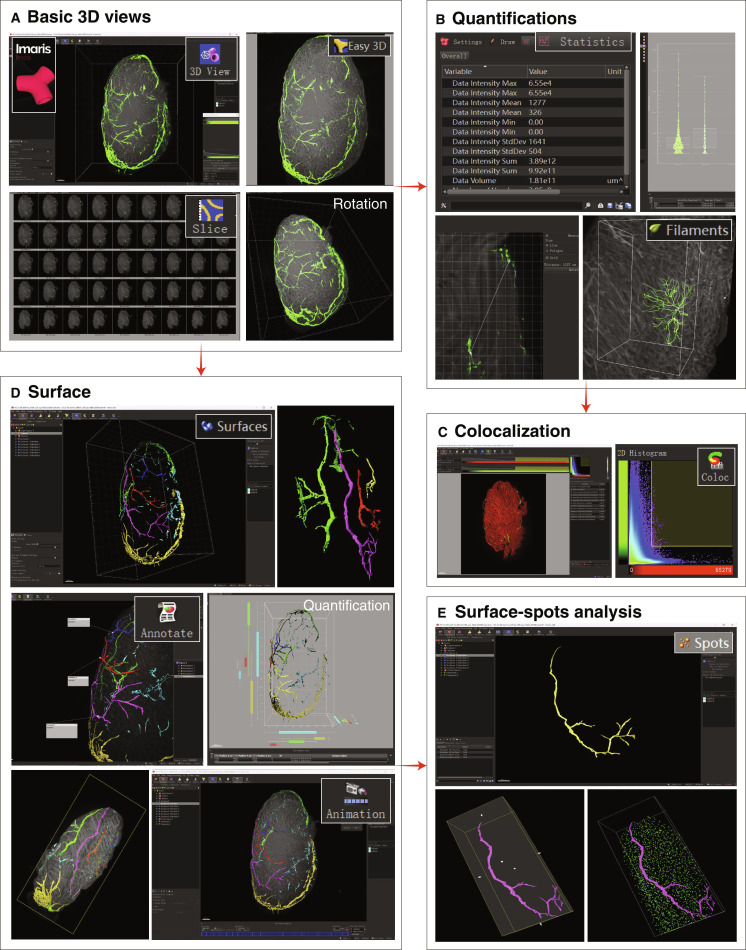
Data processing and image analysis workflow. Graphical workflow illustrating the data processing pipeline following light sheet image acquisition. Steps correspond to functions implemented in Imaris software, including 3D visualization, quantification, surface rendering, annotation, animation, colocalization, and surface–spots analysis. (A) Basic data processing using the Imaris software. (B) Data output and quantitative analysis by the statistics tool. (C) Colocalization analysis of 2 color channels. (D) 3D surface rendering of the cleared sample and various rendering modalities available in Imaris. (E) Surface-spots analysis based on the above surface rendering.

**Fig. 7.**
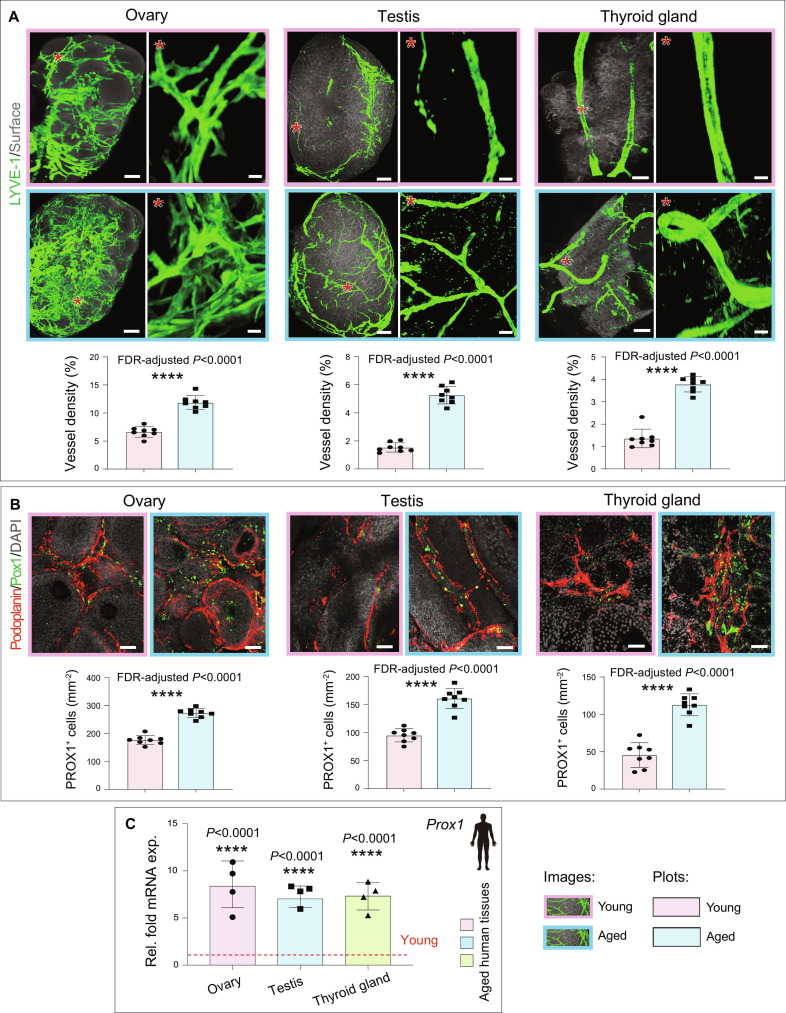
Age-associated lymphatic vessel expansion in endocrine glands. (A) Whole-organ imaging of cleared mouse ovary, testis, and thyroid gland from young and aged mice immunostained with LYVE-1. Asterisks indicate higher magnification of specific regions. Quantification of lymphatic vessel density in young and aged ovary, testis, and thyroid gland (*n* = 8). (B) 3D confocal images of young and aged ovary, testis, and thyroid gland with Podoplanin and Prox1 immunostaining. Nuclei: DAPI. Quantification of Prox1^+^ cell numbers in young and aged ovary, testis, and thyroid gland (*n* = 8). (C) qPCR analysis of *Prox1* expression (normalized to *Actb*) in aged human ovary, testis, and thyroid compared with young endocrine glands (*n* = 4). Data represent mean ± SD. *P* values were derived from 2-tailed unpaired *t* tests and corrected for multiple comparisons using the Benjamini–Hochberg false discovery rate procedure. ****P < 0.0001. Scale bars: 400 μm for whole-organ images and 50 μm for high-magnification insets (A) and 50 μm (B).

A key advantage of 3D imaging lies in its capacity to support quantitative analysis of spatial tissue organization and to reveal microenvironmental structure and function (59,60). The whole-organ imaging data obtained using this method can be processed with relevant imaging software to achieve 3D visualization, quantification, surface rendering, colocalization, animation, and other functions (Fig. 6). Next, we performed a quantitative 3D analysis of the lymphatic vessels using LYVE-1 and Prox1 immunostainings on whole organs and endocrine glands. The circulatory system encompasses both the blood vascular system and lymphatic vascular system. The lymphatic system regulates fluid homeostasis, waste clearance, and immune responses (61–63). Recent studies have revealed age-dependent changes in blood vessels, organs, and endocrine glands and have also documented the correlation of these changes with organ function (64). Here, we examined and compared the lymphatic vessels across different endocrine glands. Quantitative analysis of LYVE-1 and Prox1 immunostaining across endocrine glands from young and aged mice revealed expansion of LECs in the ovaries, testes, and thyroid glands (Fig. 7A and B). In contrast, the lymphatic vessel density in other murine organs did not show expansion with age (Fig. S8).

To understand the relevance of the age-related changes observed in these 3 murine endocrine glands, we next analyzed human endocrine tissues. Healthy human testes, ovaries, and thyroid glands from young (<20 years) and aged (>70 years) individuals (Table S2) were analyzed for the Prox1 expression by quantitative PCR (Fig. 7C). Similar to murine endocrine glands, Prox1 expression was up-regulated in aged human ovaries, testes, and thyroid glands (Fig. 7C). Together, these results further support the applicability of this method for whole-organ quantitative imaging and demonstrate its potential to capture biologically relevant changes in tissue architecture. Given the methodological focus of this study, the age-associated lymphatic alterations observed in endocrine glands are presented as an illustrative application of the approach and provide a basis for future mechanistic validation.

## Discussion

The 3D organization of functional networks, including blood and lymphatic vessels within organs, along with the spatial distribution of cells within tissues, is critical for deciphering biological complexity (10,65–67). 3D imaging of cleared large tissues or whole organs provides such information (23,66,68,69). Further, it serves as a valuable alternative to traditional histology and immunofluorescence of thin tissue sections, both of which lack the crucial 3D information (70,71). To enable rapid, simplified, and high-resolution 3D imaging of whole organs and large tissue samples, we introduce and describe this method. This approach enables 3D molecular characterization of intact organs and tissue biopsies across defined tissue regions. A sequence of tissue processing steps renders the samples susceptible to rapid and optimal immunostaining and histological staining while preserving cellular and tissue integrity. Digestion of the extracellular matrix with collagenase, together with methanol treatment, enhances antibody penetration. Collagenase-based extracellular matrix digestion, combined with antibody incubation at an elevated temperature, facilitates more rapid antibody penetration. Furthermore, these steps eliminate the need for time-consuming delipidation procedures that rely on relatively toxic organic solvents (68,72). Additionally, the bleaching step reduces light scattering (72,73). Hydrogen peroxide used for bleaching reduces tissue autofluorescence and is particularly beneficial in tissues with high heme content, such as the spleen and heart (74). Bleaching before the immunostaining helps retain fluorochromes, which otherwise are susceptible to bleaching. In addition, incubation at 37 °C was employed to facilitate antibody diffusion and improve penetration in intact tissues. Under our optimized permeabilization and washing conditions, this elevated temperature did not result in increased nonspecific staining or background signals, while it substantially improved staining uniformity across large tissue volumes. Thus, this method neither requires the injection of antibodies nor relies on transgenic mice expressing fluorescent reporters; it instead achieves multicolor immunolabeling using fluorochrome-coupled antibodies.

One limitation to note is that the biological findings presented here are primarily intended to demonstrate the analytical utility of this method. Although age-associated lymphatic expansion was observed in murine endocrine glands and was supported by increased *Prox1* expression in aged human endocrine tissues, human 3D imaging validation was not performed in the current study. Future studies incorporating 3D imaging of human endocrine tissues will be needed to further validate the relevance of these observations in human organs.

ECi and PEGM have been used independently in various clearing methods as tissue clearing components or for RI matching owing to their low cost and versatility (74,75). Here, we combined these 2, ECi and PEGM, which facilitates rapid clearing of larger tissues and organs. After clearing, images can be acquired on different imaging systems, including laser-scanning confocal microscopes for single-cell and subcellular resolution or light sheet microscopes for whole-organ overviews. Therefore, this approach enables multicolor antibody-based immunolabeling, provides high tissue transparency, and preserves cellular morphology and compatibility with multiple imaging platforms.

Recently, several methods have been developed to enable rapid tissue clearing for intact organs. Several rapid methods including the ultrafast optical clearing method (FOCM) and MACS achieve clearing in approximately 3 d but require additional time for subsequent immunostaining (76,77). The FLASH method accomplishes tissue clearing and immunolabeling within 1 to 2 weeks (38,78). These represented the fastest reported methods that enable both clearing and immunolabeling in 7 d. Compared with these methods, this method achieves 3D imaging and robust tissue clearing for multiple whole organs and large tissues in a record time of 2.5 d, thereby substantially reducing the overall processing time. Another key feature of this approach is its applicability across a broad range of murine and human tissues.

As a result of facilitating high speed, high resolution, and flexibility across organs, tissues, fluorochromes, and imaging platforms, this method has the potential to render tissue clearing and 3D imaging more widely accessible to a broader audience. It is a versatile technique capable of addressing a wide variety of questions throughout the biological sciences. Thus, this approach expands the repertoire of tools and knowledge available for investigating complex biological processes that require spatial and architectural knowledge of cells within tissues and organs.

## Conclusion

In this study, we established a rapid and streamlined 3D imaging workflow that achieves multicolor immunolabeling, deep antibody penetration, and efficient clearing of whole organs within approximately 2.5 d. The method preserves tissue morphology and endogenous fluorescence while supporting robust quantitative analyses across diverse murine and human tissues. By avoiding highly toxic organic solvents and substantially reducing overall processing time, this approach broadens the accessibility of whole-organ 3D imaging and provides a powerful platform for investigating tissue architecture, vascular and lymphatic organization, as well as age- and disease-associated changes within intact organs.

## Data Availability

Data presented in this study are available upon request from the corresponding author.

## References

[1] Belle M, Godefroy D, Couly G, Malone SA, Collier F, Giacobini P, Chédotal A. Tridimensional visualization and analysis of early human development. Cell. 2017;169(1):161–173.e12. 28340341 10.1016/j.cell.2017.03.008

[2] Ding Y, Ma J, Langenbacher AD, Baek KI, Lee J, Chang CC, Hsu JJ, Kulkarni RP, Belperio J, Shi W, et al. Multiscale light-sheet for rapid imaging of cardiopulmonary system. JCI Insight. 2018;3(16): Article e121396. 30135307 10.1172/jci.insight.121396 PMC6141183

[3] Udan RS, Piazza VG, Hsu CW, Hadjantonakis A-K, Dickinson ME. Quantitative imaging of cell dynamics in mouse embryos using light-sheet microscopy. Development. 2014;141(22):4406–4414. 25344073 10.1242/dev.111021 PMC4302910

[4] Keller PJ, Schmidt AD, Wittbrodt J, Stelzer EH. Reconstruction of zebrafish early embryonic development by scanned light sheet microscopy. Science. 2008;322(5904):1065–1069. 18845710 10.1126/science.1162493

[5] Takebe T, Sekine K, Kimura M, Yoshizawa E, Ayano S, Koido M, Funayama S, Nakanishi N, Hisai T, Kobayashi T, et al. Massive and reproducible production of liver buds entirely from human pluripotent stem cells. Cell Rep. 2017;21(10):2661–2670. 29212014 10.1016/j.celrep.2017.11.005

[6] Huisken J, Swoger J, Del Bene F, Wittbrodt J, Stelzer EH. Optical sectioning deep inside live embryos by selective plane illumination microscopy. Science. 2004;305(5686):1007–1009. 15310904 10.1126/science.1100035

[7] Shi MY, Yao Y, Wang M, Yang Q, Ding L, Li R, Li Y, Huang H, Yang CY, Zhou Z, et al. High-speed mapping of whole-mouse peripheral nerves at subcellular resolution. Cell. 2025;188(14):3897–3915.20. 40645171 10.1016/j.cell.2025.06.011

[8] Ou Z, Duh YS, Rommelfanger NJ, Keck CHC, Jiang S, Brinson K Jr, Zhao S, Schmidt EL, Wu X, Yang F, et al. Achieving optical transparency in live animals with absorbing molecules. Science. 2024;385(6713): Article eadm6869. 39236186 10.1126/science.adm6869 PMC11931656

[9] Wolff C, Tinevez JY, Pietzsch T, Stamataki E, Harich B, Guignard L, Preibisch S, Shorte S, Keller PJ, Tomancak P, et al. Multi-view light-sheet imaging and tracking with the MaMuT software reveals the cell lineage of a direct developing arthropod limb. Elife. 2018;7: Article e34410. 29595475 10.7554/eLife.34410 PMC5929908

[10] Singh A, Veeriah V, Xi P, Labella R, Chen J, Romeo SG, Ramasamy SK, Kusumbe AP. Angiocrine signals regulate quiescence and therapy resistance in bone metastasis. JCI Insight. 2019;4(13): Article e125679. 31292293 10.1172/jci.insight.125679 PMC6629249

[11] Klingberg A, Hasenberg A, Ludwig-Portugall I, Medyukhina A, Männ L, Brenzel A, Engel DR, Figge MT, Kurts C, Gunzer M. Fully automated evaluation of total glomerular number and capillary tuft size in nephritic kidneys using lightsheet microscopy. J Am Soc Nephrol. 2017;28(2):452–459. 27487796 10.1681/ASN.2016020232 PMC5280021

[12] Yi Y, Li Y, Zhang S, Men Y, Wang Y, Jing D, Ding J, Zhu Q, Chen Z, Chen X, et al. Mapping of individual sensory nerve axons from digits to spinal cord with the transparent embedding solvent system. Cell Res. 2024;34(2):124–139. 38168640 10.1038/s41422-023-00867-3 PMC10837210

[13] Stucker S, De Angelis J, Kusumbe AP. Heterogeneity and dynamics of vasculature in the endocrine system during aging and disease. Front Physiol. 2021;12: Article 624928. 33767633 10.3389/fphys.2021.624928 PMC7987104

[14] Liu H, Liu L, Zhang Y, Tian Q, Ding Z, Chen J, Kusumbe AP. Lymphatic-immune interactions in the musculoskeletal system. Front Immunol. 2025;16:1578847. 40496852 10.3389/fimmu.2025.1578847 PMC12148863

[15] Kumar N, Saraber P, Ding Z, Kusumbe AP. Diversity of vascular niches in bones and joints during homeostasis, ageing, and diseases. Front Immunol. 2021;12: Article 798211. 34975909 10.3389/fimmu.2021.798211 PMC8718446

[16] Fan Y, Elkhalek M, Zhang Y, Liu L, Tian Q, Chueakula N, Ramasamy SK, Dalan R, Habib SJ, Kusumbe AP. Bone marrow adipocytes: Key players in vascular niches, aging, and disease. Front Cell Dev Biol. 2025;13:1633801. 40852589 10.3389/fcell.2025.1633801 PMC12367753

[17] Chen J, Hendriks M, Chatzis A, Ramasamy SK, Kusumbe AP. Bone vasculature and bone marrow vascular niches in health and disease. J Bone Miner Res. 2020;35(11):2103–2120. 32845550 10.1002/jbmr.4171

[18] Li L, Rottmann I, Saeed BR, Ivison G, Wei H, Schroeder JC, Dunkel G, Greif K, Zhang Y, Makky A, et al. High-dimensional spatiotemporal single-cell atlas and 3D imaging of bone marrow microenvironment during CML progression. Blood. 2026; Article blood-2025029824.10.1182/blood.202502982441592325

[19] Yang Y, Fan Y, Jain S, Ding Z, Liu H, Horenberg AL, Sun H, Killer MS, Chandra A, Dalan R, et al. Degeneration and impaired resilience of skull bone and hematopoietic bone marrow. bioRxiv. 2025. 10.1101/2025.10.02.679940

[20] Biswas L, Chen J, De Angelis J, Singh A, Owen-Woods C, Ding Z, Pujol JM, Kumar N, Zeng F, Ramasamy SK, et al. Lymphatic vessels in bone support regeneration after injury. Cell. 2023;186(2):382–397.e24. 36669473 10.1016/j.cell.2022.12.031 PMC11913777

[21] Zhang Y, Tian Q, Yang Y, Liu H, Yesin TK, Lu W, Joseph JD, Borah B, Ramasamy S, Yun MH, et al. From repair to disease: Lymphatic contributions to regeneration, cancer and ageing. J Adv Res. 2026. 10.1016/j.jare.2026.04.005 41946393

[22] Chen BC, Legant WR, Wang K, Shao L, Milkie DE, Davidson MW, Janetopoulos C, Wu XS, Hammer JA III, Liu Z, et al. Lattice light-sheet microscopy: Imaging molecules to embryos at high spatiotemporal resolution. Science. 2014;346(6208): Article 1257998. 25342811 10.1126/science.1257998 PMC4336192

[23] Susaki EA, Ueda HR. Whole-body and whole-organ clearing and imaging techniques with single-cell resolution: Toward organism-level systems biology in mammals. Cell Chem Biol. 2016;23(1):137–157. 26933741 10.1016/j.chembiol.2015.11.009

[24] Zhao S, Todorov MI, Cai R, Ai-Maskari RA, Steinke H, Kemter E, Mai H, Rong Z, Warmer M, Stanic K, et al. Cellular and molecular probing of intact human organs. Cell. 2020;180(4):796–812.e19. 32059778 10.1016/j.cell.2020.01.030 PMC7557154

[25] Ni Y, Wu J, Liu F, Yi Y, Meng X, Gao X, Xiao L, Zhou W, Chen Z, Chu P, et al. Deep imaging of LepR^+^ stromal cells in optically cleared murine bone hemisections. Bone Res. 2025;13(1):6. 39800733 10.1038/s41413-024-00387-9 PMC11725602

[26] Kunz L, Schroeder T. A 3D tissue-wide digital imaging pipeline for quantitation of secreted molecules shows absence of CXCL12 gradients in bone marrow. Cell Stem Cell. 2019;25(6):846–854.e4. 31809740 10.1016/j.stem.2019.10.003

[27] Dettinger P, Kull T, Arekatla G, Ahmed N, Zhang Y, Schneiter F, Wehling A, Schirmacher D, Kawamura S, Loeffler D, et al. Open-source personal pipetting robots with live-cell incubation and microscopy compatibility. Nat Commun. 2022;13(1):2999. 35637179 10.1038/s41467-022-30643-7 PMC9151679

[28] Reismann D, Stefanowski J, Günther R, Rakhymzhan A, Matthys R, Nützi R, Zehentmeier S, Schmidt-Bleek K, Petkau G, Chang HD, et al. Longitudinal intravital imaging of the femoral bone marrow reveals plasticity within marrow vasculature. Nat Commun. 2017;8(1):2153. 29255233 10.1038/s41467-017-01538-9 PMC5735140

[29] Keller PJ, editor. *Reconstructing nervous system development and function with light-sheet microscopy*. San Jose (CA): CLEO; 2025.

[30] Prahst C, Ashrafzadeh P, Mead T, Figueiredo A, Chang K, Richardson D, Venkaraman L, Richards M, Russo AM, Harrington K, et al. Mouse retinal cell behaviour in space and time using light sheet fluorescence microscopy. Elife. 2020;9: Article e49779. 32073398 10.7554/eLife.49779 PMC7162655

[31] Todorov MI, Paetzold JC, Schoppe O, Tetteh G, Shit S, Efremov V, Todorov-Völgyi K, Düring M, Dichgans M, Piraud M, et al. Machine learning analysis of whole mouse brain vasculature. Nat Methods. 2020;17(4):442–449. 32161395 10.1038/s41592-020-0792-1 PMC7591801

[32] Li C, Li Y, Zhao H, Ding L. Enhancing brain image quality with 3D U-net for stripe removal in light sheet fluorescence microscopy. Brain Inform. 2024;11(1):24. 39325110 10.1186/s40708-024-00236-9 PMC11427638

[33] Coutu DL, Kokkaliaris KD, Kunz L, Schroeder T. Multicolor quantitative confocal imaging cytometry. Nat Methods. 2018;15(1):39–46. 29320487 10.1038/nmeth.4503

[34] McDonald MM, Khoo WH, Ng PY, Xiao Y, Zamerli J, Thatcher P, Kyaw W, Pathmanandavel K, Grootveld AK, Moran I, et al. Osteoclasts recycle via osteomorphs during RANKL-stimulated bone resorption. Cell. 2021;184(5):1330–1347.e13. 33636130 10.1016/j.cell.2021.02.002 PMC7938889

[35] Wang K, Xie Y, Moore D, Houchen C, Lu Y, Ray E, Dallas M. Tissue expression of GFP-tagged collagen in transgenic mice and live cell imaging of osteoblast collagen assembly and bone collagen resorption. In: *ASBMR Annual Meeting*. Washington (DC): ASBMR; 2024.

[36] Tissot FS, Gonzalez-Anton S, Lo Celso C. Intravital microscopy to study the effect of matrix metalloproteinase inhibition on acute myeloid leukemia cell migration in the bone marrow. Methods Mol Biol. 2024;2747:211–227. 38038943 10.1007/978-1-0716-3589-6_17

[37] Huang Q, Cohen MA, Alsina FC, Devlin G, Garrett A, McKey J, Havlik P, Rakhilin N, Wang E, Xiang K, et al. Intravital imaging of mouse embryos. Science. 2020;368(6487):181–186. 32273467 10.1126/science.aba0210 PMC7646360

[38] Messal HA, Almagro J, Zaw Thin M, Tedeschi A, Ciccarelli A, Blackie L, Anderson KI, Miguel-Aliaga I, van Rheenen J, Behrens A. Antigen retrieval and clearing for whole-organ immunofluorescence by FLASH. Nat Protoc. 2021;16(1):239–262. 33247285 10.1038/s41596-020-00414-z

[39] Sato Y, Miyawaki T, Ouchi A, Noguchi A, Yamaguchi S, Ikegaya Y. Quick visualization of neurons in brain tissues using an optical clearing technique. Anat Sci Int. 2019;94(2):199–208. 30600446 10.1007/s12565-018-00473-z

[40] Power RM, Huisken J. A guide to light-sheet fluorescence microscopy for multiscale imaging. Nat Methods. 2017;14(4):360–373. 28362435 10.1038/nmeth.4224

[41] Matryba P, Kaczmarek L, Gołąb J. Advances in ex situ tissue optical clearing. Laser Photonics Rev. 2019;13(8):1800292.

[42] Yu T, Zhu J, Li D, Zhu D. Physical and chemical mechanisms of tissue optical clearing. iScience. 2021;24(3): Article 102178. 33718830 10.1016/j.isci.2021.102178 PMC7920833

[43] Oliveira LMC, Tuchin VV. *The optical clearing method: A new tool for clinical practice and biomedical engineering*. Berlin (Germany): Springer Nature; 2019.

[44] Mai H, Luo J, Hoeher L, Al-Maskari R, Horvath I, Chen Y, Kofler F, Piraud M, Paetzold JC, Modamio J, et al. Whole-body cellular mapping in mouse using standard IgG antibodies. Nat Biotechnol. 2024;42(4):617–627. 37430076 10.1038/s41587-023-01846-0 PMC11021200

[45] Otomo K, Omura T, Nozawa Y, Edwards SJ, Sato Y, Saito Y, Yagishita S, Uchida H, Watakabe Y, Naitou K, et al. descSPIM: An affordable and easy-to-build light-sheet microscope optimized for tissue clearing techniques. Nat Commun. 2024;15(1):4941. 38866781 10.1038/s41467-024-49131-1 PMC11169475

[46] Grüneboom A, Hawwari I, Weidner D, Culemann S, Müller S, Henneberg S, Brenzel A, Merz S, Bornemann L, Zec K, et al. A network of trans-cortical capillaries as mainstay for blood circulation in long bones. Nat Metab. 2019;1(2):236–250. 31620676 10.1038/s42255-018-0016-5 PMC6795552

[47] Matsumoto K, Mitani TT, Horiguchi SA, Kaneshiro J, Murakami TC, Mano T, Fujishima H, Konno A, Watanabe TM, Hirai H, et al. Advanced CUBIC tissue clearing for whole-organ cell profiling. Nat Protoc. 2019;14(12):3506–3537. 31748753 10.1038/s41596-019-0240-9

[48] Hama H, Hioki H, Namiki K, Hoshida T, Kurokawa H, Ishidate F, Kaneko T, Akagi T, Saito T, Saido T, et al. ScaleS: An optical clearing palette for biological imaging. Nat Neurosci. 2015;18(10):1518–1529. 26368944 10.1038/nn.4107

[49] Jing D, Zhang S, Luo W, Gao X, Men Y, Ma C, Liu X, Yi Y, Bugde A, Zhou BO, et al. Tissue clearing of both hard and soft tissue organs with the PEGASOS method. Cell Res. 2018;28(8):803–818. 29844583 10.1038/s41422-018-0049-z PMC6082844

[50] Azaripour A, Lagerweij T, Scharfbillig C, Jadczak AE, Willershausen B, Van Noorden CJF. A survey of clearing techniques for 3D imaging of tissues with special reference to connective tissue. Prog Histochem Cytochem. 2016;51(2):9–23. 27142295 10.1016/j.proghi.2016.04.001

[51] Chung K, Wallace J, Kim SY, Kalyanasundaram S, Andalman AS, Davidson TJ, Mirzabekov JJ, Zalocusky KA, Mattis J, Denisin AK, et al. Structural and molecular interrogation of intact biological systems. Nature. 2013;497(7449):332–337. 23575631 10.1038/nature12107 PMC4092167

[52] Renier N, Wu Z, Simon DJ, Yang J, Ariel P, Tessier-Lavigne M. iDISCO: A simple, rapid method to immunolabel large tissue samples for volume imaging. Cell. 2014;159(4):896–910. 25417164 10.1016/j.cell.2014.10.010

[53] Murray E, Cho JH, Goodwin D, Ku T, Swaney J, Kim SY, Choi H, Park YG, Park JY, Hubbert A, et al. Simple, scalable proteomic imaging for high-dimensional profiling of intact systems. Cell. 2015;163(6):1500–1514. 26638076 10.1016/j.cell.2015.11.025 PMC5275966

[54] Ertürk A, Becker K, Jährling N, Mauch CP, Hojer CD, Egen JG, Hellal F, Bradke F, Sheng M, Dodt HU. Three-dimensional imaging of solvent-cleared organs using 3DISCO. Nat Protoc. 2012;7(11):1983–1995. 23060243 10.1038/nprot.2012.119

[55] Dodt HU, Leischner U, Schierloh A, Jährling N, Mauch CP, Deininger K, Deussing JM, Eder M, Zieglgänsberger W, Becker K. Ultramicroscopy: Three-dimensional visualization of neuronal networks in the whole mouse brain. Nat Methods. 2007;4(4):331–336. 17384643 10.1038/nmeth1036

[56] Ema M, Takahashi S, Rossant J. Deletion of the selection cassette, but not cis-acting elements, in targeted Flk1-lacZ allele reveals Flk1 expression in multipotent mesodermal progenitors. Blood. 2006;107(1):111–117. 16166582 10.1182/blood-2005-05-1970

[57] Madisen L, Zwingman TA, Sunkin SM, Oh SW, Zariwala HA, Gu H, Ng LL, Palmiter RD, Hawrylycz MJ, Jones AR, et al. A robust and high-throughput Cre reporting and characterization system for the whole mouse brain. Nat Neurosci. 2010;13(1):133–140. 20023653 10.1038/nn.2467 PMC2840225

[58] Ludin A, Itkin T, Gur-Cohen S, Mildner A, Shezen E, Golan K, Kollet O, Kalinkovich A, Porat Z, D’uva G, et al. Monocytes-macrophages that express α-smooth muscle actin preserve primitive hematopoietic cells in the bone marrow. Nat Immunol. 2012;13(11):1072–1082. 22983360 10.1038/ni.2408

[59] Wu Q, Zhang J, Kumar S, Shen S, Kincaid M, Johnson CB, Zhang YS, Turcotte R, Alt C, Ito K, et al. Resilient anatomy and local plasticity of naive and stress haematopoiesis. Nature. 2024;627(8005):839–846. 38509363 10.1038/s41586-024-07186-6 PMC10972750

[60] Nombela-Arrieta C, Pivarnik G, Winkel B, Canty KJ, Harley B, Mahoney JE, Park SY, Lu J, Protopopov A, Silberstein LE. Quantitative imaging of haematopoietic stem and progenitor cell localization and hypoxic status in the bone marrow microenvironment. Nat Cell Biol. 2013;15(5):533–543. 23624405 10.1038/ncb2730 PMC4156024

[61] Alitalo K. The lymphatic vasculature in disease. Nat Med. 2011;17(11):1371–1380. 22064427 10.1038/nm.2545

[62] Davis MJ, Zawieja SD, King PD. Transport and immune functions of the lymphatic system. Annu Rev Physiol. 2025;87(1):151–172. 39441893 10.1146/annurev-physiol-022724-104908

[63] Liu L, Zhang Y, Liu H, Yang J, Tian Q, Chueakula N, Ramasamy S, Verma NK, Cheung C, Kusumbe AP. Transforming cancer therapy: Unlocking the potential of targeting vascular and stromal cells in the tumor microenvironment. Cancer Res. 2025;85(12):2152–2164. 40173050 10.1158/0008-5472.CAN-24-4744 PMC12167934

[64] Chen J, Lippo L, Labella R, Tan SL, Marsden BD, Dustin ML, Ramasamy SK, Kusumbe AP. Decreased blood vessel density and endothelial cell subset dynamics during ageing of the endocrine system. EMBO J. 2021;40(1): Article e105242. 33215738 10.15252/embj.2020105242 PMC7780152

[65] Xiao Z, Dai Z, Locasale JW. Metabolic landscape of the tumor microenvironment at single cell resolution. Nat Commun. 2019;10(1):3763. 31434891 10.1038/s41467-019-11738-0 PMC6704063

[66] Tanaka N, Kanatani S, Tomer R, Sahlgren C, Kronqvist P, Kaczynska D, Louhivuori L, Kis L, Lindh C, Mitura P, et al. Whole-tissue biopsy phenotyping of three-dimensional tumours reveals patterns of cancer heterogeneity. Nat Biomed Eng. 2017;1(10):796–806. 31015588 10.1038/s41551-017-0139-0

[67] Zhu E, Zhang Y, Zhao P, Cho JM, Wang Z, Li YR, Wang J, Margolis S, Wang S, Yang L, et al. Refractive index-corrected light-sheet microscopy for macro-view cardiovascular imaging. Adv Sci. 2025;12(38): Article e03684. 10.1002/advs.202503684 PMC12520573 40653746

[68] Lee E, Choi J, Jo Y, Kim JY, Jang YJ, Lee HM, Kim SY, Lee HJ, Cho K, Jung N, et al. ACT-PRESTO: Rapid and consistent tissue clearing and labeling method for 3-dimensional (3D) imaging. Sci Rep. 2016;6(1):18631. 26750588 10.1038/srep18631 PMC4707495

[69] Zhu J, Liu X, Liu Z, Deng Y, Xu J, Liu K, Zhang R, Meng X, Fei P, Yu T, et al. SOLID: Minimizing tissue distortion for brain-wide profiling of diverse architectures. Nat Commun. 2024;15(1):8303. 39333107 10.1038/s41467-024-52560-7 PMC11436996

[70] Uhlén P, Tanaka N. Improved pathological examination of tumors with 3D light-sheet microscopy. Trends Cancer. 2018;4(5):337–341. 29709257 10.1016/j.trecan.2018.03.003

[71] Glaser AK, Reder NP, Chen Y, McCarty EF, Yin C, Wei L, Wang Y, True LD, Liu JT. Light-sheet microscopy for slide-free non-destructive pathology of large clinical specimens. Nat Biomed Eng. 2017;1(7):0084. 29750130 10.1038/s41551-017-0084 PMC5940348

[72] Ueda HR, Ertürk A, Chung K, Gradinaru V, Chédotal A, Tomancak P, Keller PJ. Tissue clearing and its applications in neuroscience. Nat Rev Neurosci. 2020;21(2):61–79. 31896771 10.1038/s41583-019-0250-1 PMC8121164

[73] Susaki EA, Shimizu C, Kuno A, Tainaka K, Li X, Nishi K, Morishima K, Ono H, Ode KL, Saeki Y, et al. Versatile whole-organ/body staining and imaging based on electrolyte-gel properties of biological tissues. Nat Commun. 2020;11(1):1982. 32341345 10.1038/s41467-020-15906-5 PMC7184626

[74] Merz SF, Korste S, Bornemann L, Michel L, Stock P, Squire A, Soun C, Engel DR, Detzer J, Lörchner H, et al. Contemporaneous 3D characterization of acute and chronic myocardial I/R injury and response. Nat Commun. 2019;10(1):2312. 31127113 10.1038/s41467-019-10338-2 PMC6534576

[75] Masselink W, Reumann D, Murawala P, Pasierbek P, Taniguchi Y, Bonnay F, Meixner K, Knoblich JA, Tanaka EM. Broad applicability of a streamlined ethyl cinnamate-based clearing procedure. Development. 2019;146(3): Article dev166884. 30665888 10.1242/dev.166884 PMC7115989

[76] Zhu X, Huang L, Zheng Y, Song Y, Xu Q, Wang J, Si K, Duan S, Gong W. Ultrafast optical clearing method for three-dimensional imaging with cellular resolution. Proc Natl Acad Sci USA. 2019;116(23):11480–11489. 31101714 10.1073/pnas.1819583116 PMC6561250

[77] Zhu J, Yu T, Li Y, Xu J, Qi Y, Yao Y, Ma Y, Wan P, Chen Z, Li X, et al. MACS: Rapid aqueous clearing system for 3D mapping of intact organs. Adv Sci. 2020;7(8):1903185. 10.1002/advs.201903185 PMC7175264 32328422

[78] Li W, Germain RN, Gerner MY. High-dimensional cell-level analysis of tissues with Ce3D multiplex volume imaging. Nat Protoc. 2019;14(6):1708–1733. 31028373 10.1038/s41596-019-0156-4 PMC8690297

