# Supplementary material for: Rapid 3D Immunolabeling and Light Sheet Microscopy for Quantitative Analysis of Intact Tissues
Source: Comput Struct Biotechnol J. 2026 May 21;35(1):0121. doi: 10.34133/csbj.0121 (PMC13191089; doi:10.34133/csbj.0121)
Supplement: Supplementary 1 — Figs. S1 to S8 Tables S1 to S4 Movies S1 to S5 [file csbj.0121.f1.zip › Supplementary information.pdf]

Supporting Information

Rapid 3D Immunolabeling and Light Sheet Microscopy for Quantitative Analysis of Intact Tissues

Supplementary Figures

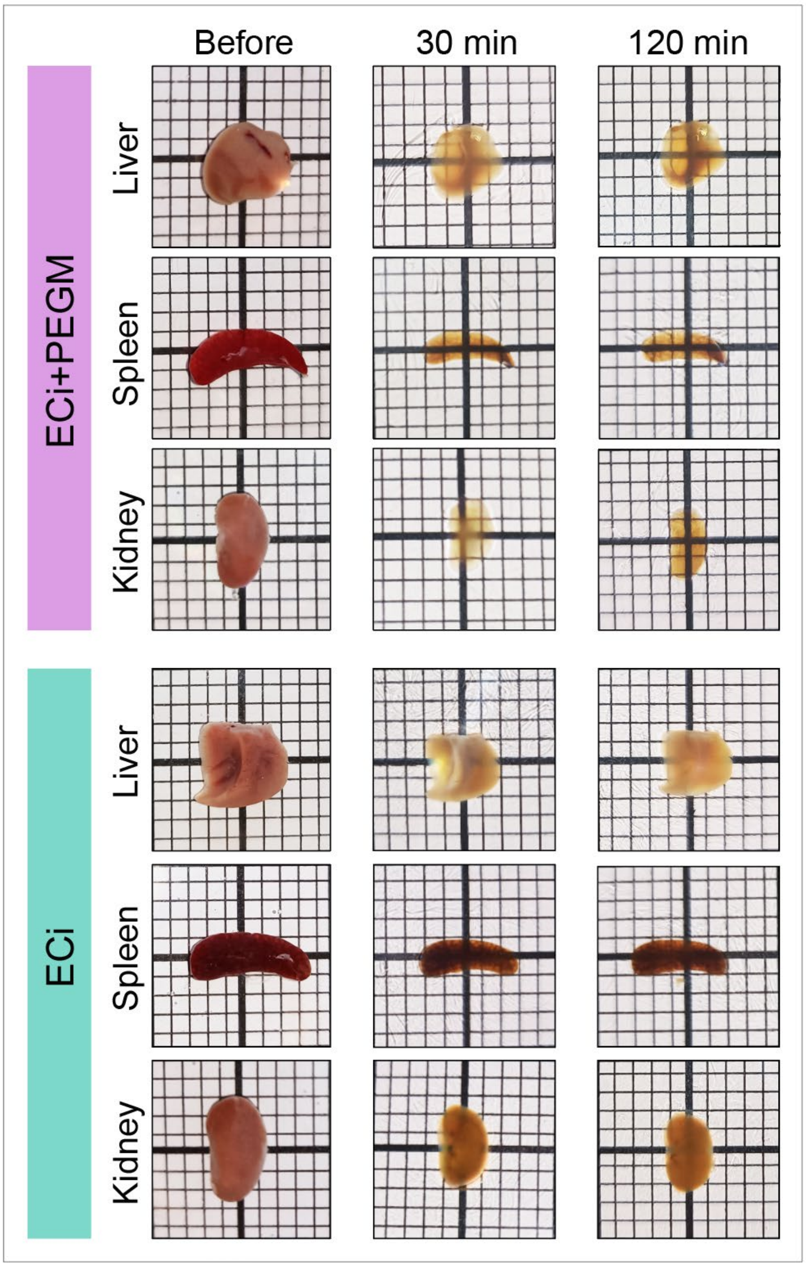

Figure S1. Comparison of tissue clearing efficiency following treatment with ECi+PEGM and ECi. Representative photos of mouse liver, spleen, and kidney before clearing and after 30 and 120 min of treatment.

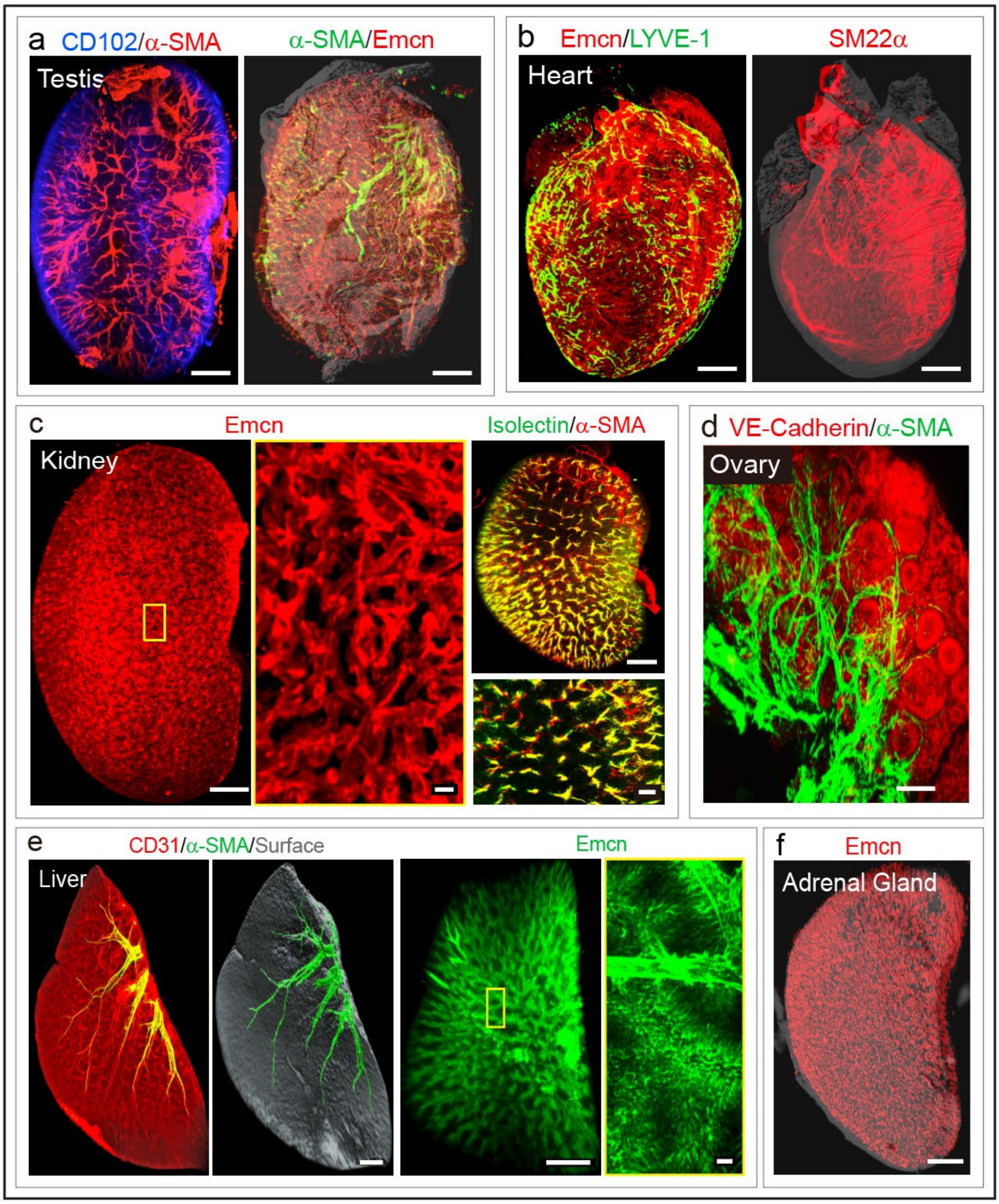

Figure S2. Light-sheet imaging of whole cleared mouse organs and endocrine glands. a) Representative 3D images acquired on a light sheet microscope of whole cleared testis stained with the antibodies indicated in the panel. **b)** Whole-organ imaging of cleared mouse heart stained with Emcn, LYVE-1 and SM22α. **c)** Representative 3D images of cleared kidney, with inset showing high magnification of specific region. **d)** 3D image of whole cleared ovary stained with VE-Cadherin and α-SMA. **e)** Representative 3D imaging of whole cleared liver immuno-stained with CD31, α-SMA and Emcn. Inset shows high magnification region. **f)** Whole-organ imaging of cleared adrenal gland stained with Emcn. Scale bars are 400 μm (a, b

and d); Scale bar is 200 μm (f); Scale bars are 400 μm for whole-organ images and 50 μm for the high magnification insets (c, e).

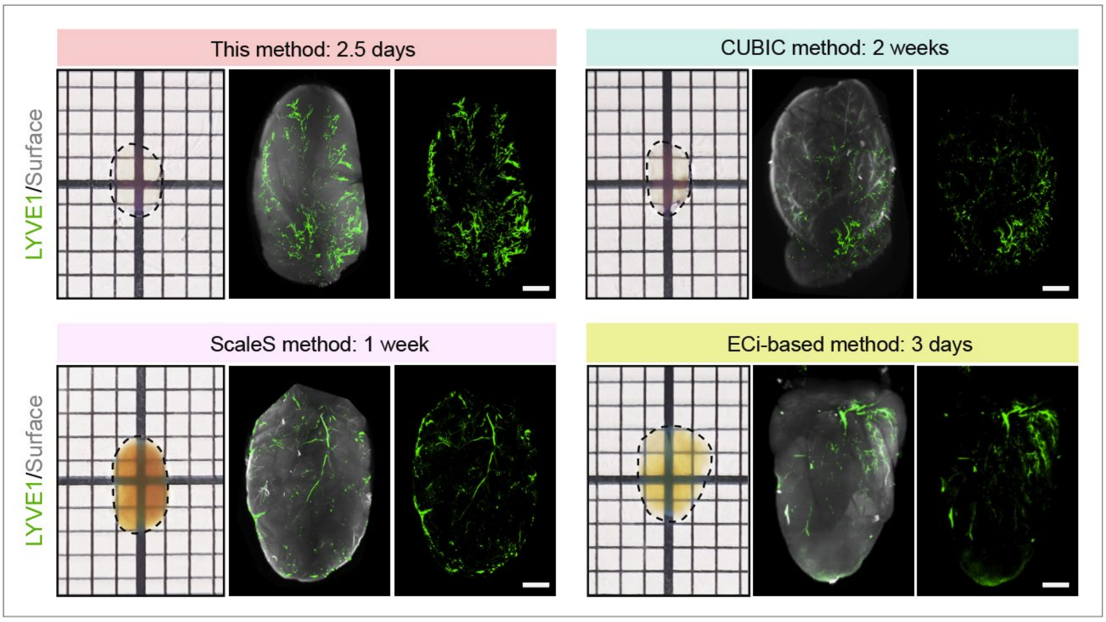

Figure S3. Representative photographs and light-sheet 3D images of mouse salivary glands immunostained for LYVE1 processed using four tissue clearing methods including this method, CUBIC, ScaleS, and ECi-based approaches. Scale bars: 1000 μm.

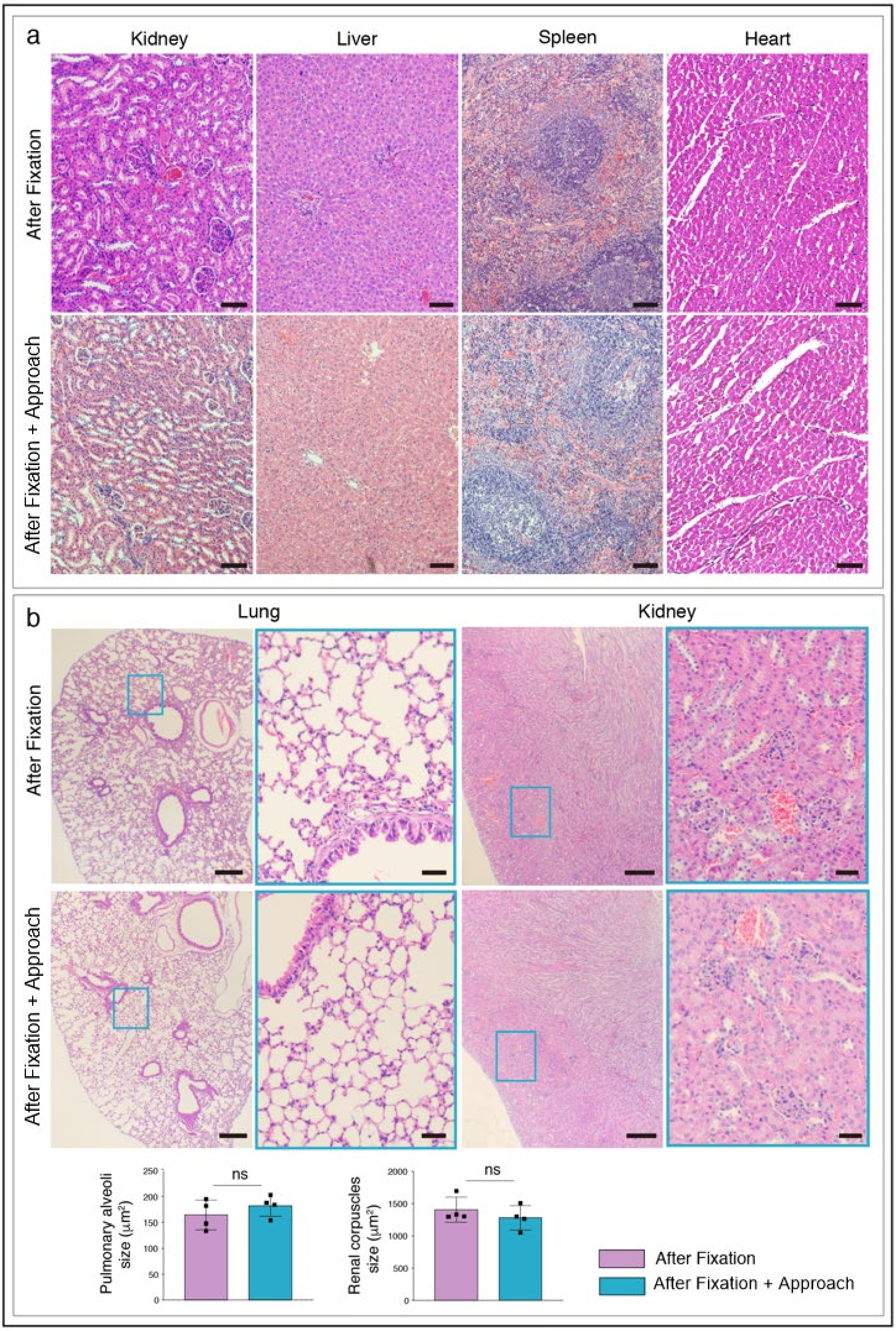

Figure S4. This method preserves tissue integrity, and the tissues can be used for histology. **a)** H&E staining of mouse kidney, liver, spleen and heart after fixation (up) and fixation followed by this imaging approach (below). **b)** H&E staining of mouse lung and Kidney after

fixation and fixation followed by these processes. Inset shows high magnification of specific region. Bar graph (left) shows quantifications of pulmonary alveoli size in lung after fixation and after fixation followed by processes of this method (*n=4*). Bar graph (right) shows quantifications of renal corpuscles size in kidney (*n=4*). Data represents mean±s.d., p-value derived from two-tailed unpaired *t*-tests is given for all graphs. ns, not significant. Scale bars are 200 μm (a). Scale bars are 400 μm for images and 100 μm for the high magnification insets (b).

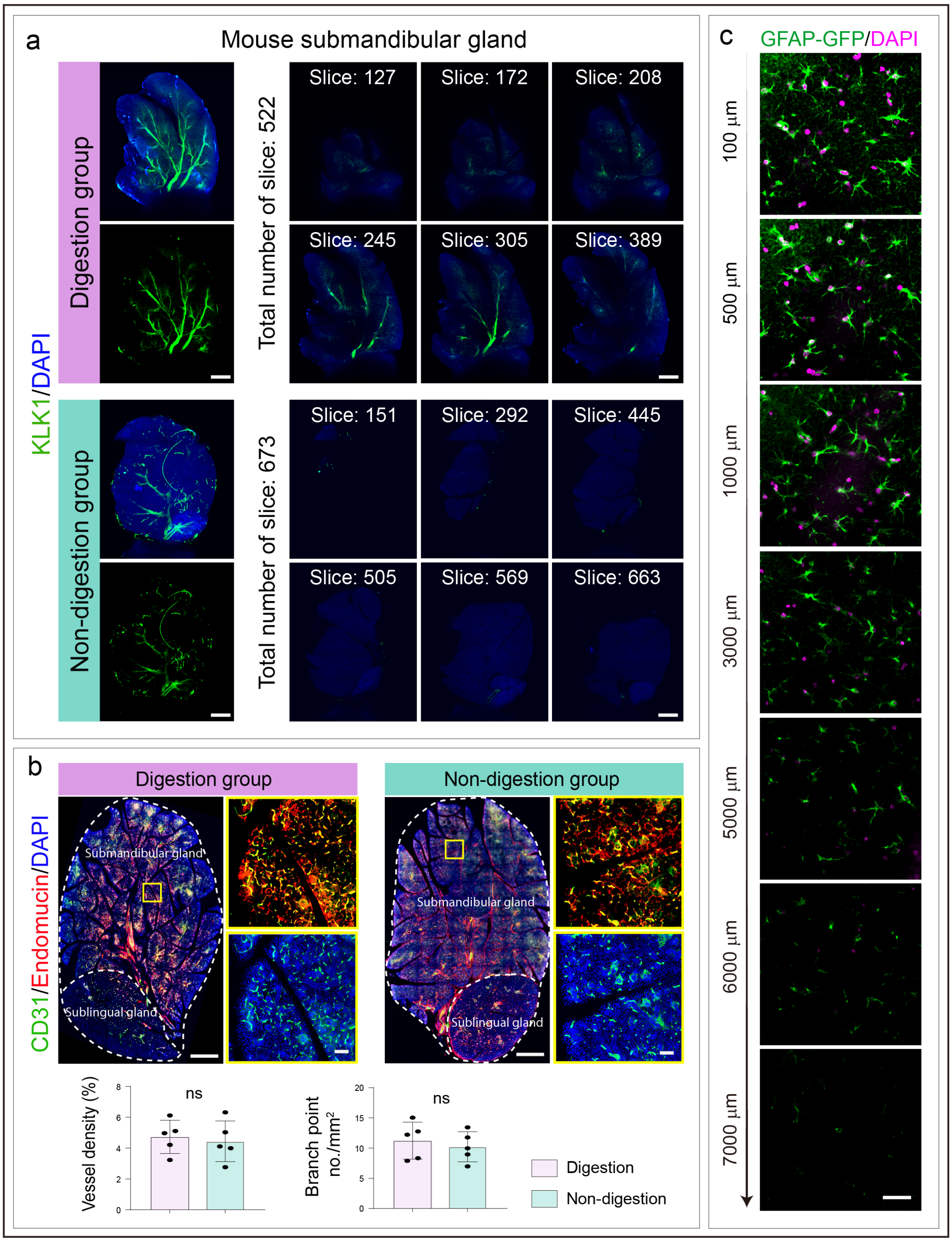

Figure S5. **a)** Representative images of mouse submandibular glands with or without collagenase A digestion, showing the ductal system immunostained for KLK1. The slice views gallery shows the representative longitudinal sections across the entire glands. Scale bars: 800 µm. **b)** Representative confocal 3D images of mouse salivary glands processed by the collagenase digestion method and the non-digestion method. Quantifications of vessel density and branch point number. Scale bars: 1000 µm, insets: 50 µm. ns: no significance. **c)** Representative light-sheet images of cleared *GFAP-GFP* mouse brain showing GFAP^{+} astrocytes (green) and DAPI (magenta) at increasing depths (100–7000 µm). Scale bar: 200 µm.

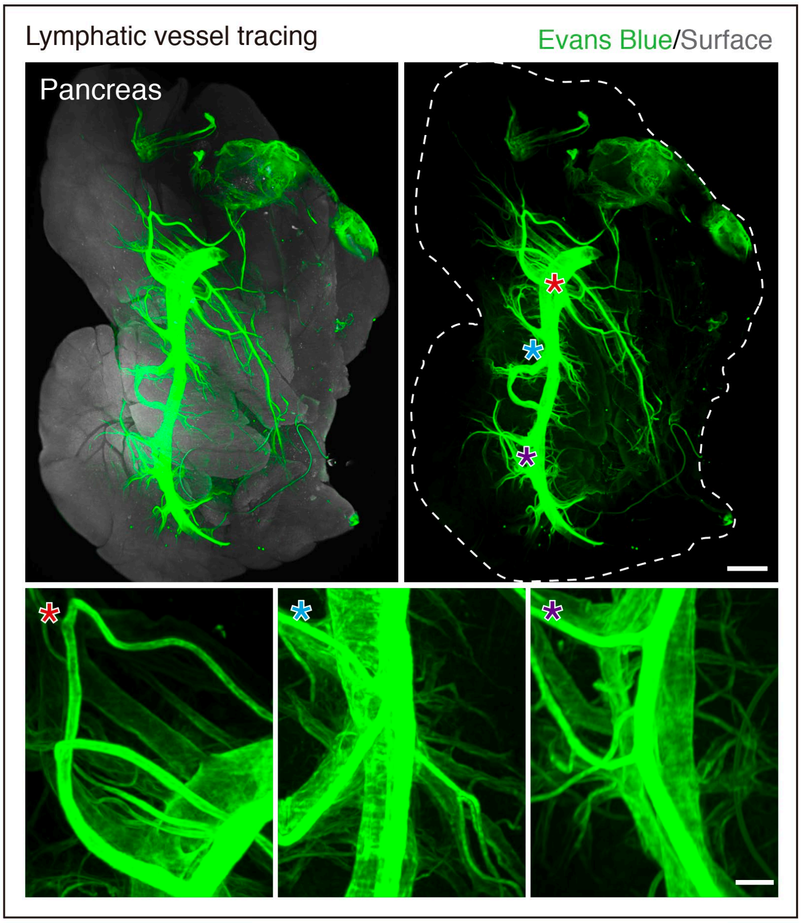

Figure S6. 3D imaging of lymphatic vessel tracing. Representative 3D images of cleared whole mouse pancreas after the injection of Evans blue dye into the inner leg, medial to the tail, and footpad. Insets (asterisk) show high magnification of lymphatic vessels in specific regions. Scale bars are 400 µm for images and 100 µm for the high magnification insets.

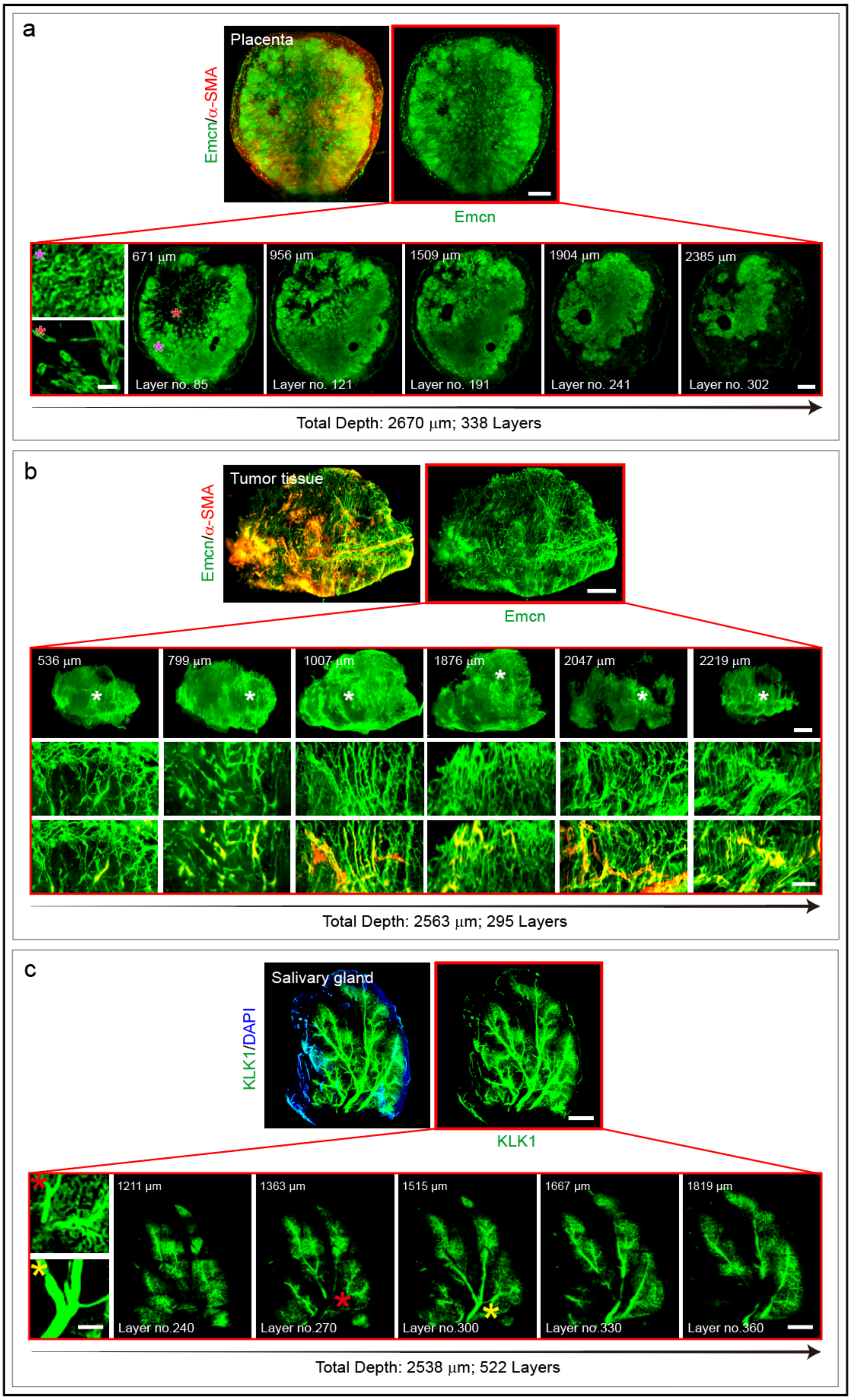
a
Placenta
Emcn/α-SMA
Emcn
671 μm
956 μm
1509 μm
1904 μm
2385 μm
Layer no. 85
Layer no. 121
Layer no. 191
Layer no. 241
Layer no. 302
Total Depth: 2670 μm; 338 Layers
b
Tumor tissue
Emcn/α-SMA
Emcn
536 μm
799 μm
1007 μm
1876 μm
2047 μm
2219 μm
Total Depth: 2563 μm; 295 Layers
c
Salivary gland
KLK1/DAPI
KLK1
1211 μm
1363 μm
1515 μm
1667 μm
1819 μm
Layer no.240
Layer no.270
Layer no.300
Layer no.330
Layer no.360
Total Depth: 2538 μm; 522 Layers

Figure S7. Imaging depth in whole-organ imaging of cleared mouse organs using this method. a) Whole-organ imaging (top) of cleared mouse placenta stained with Emcn and α-SMA acquired on a light-sheet microscope. The representative section gallery views (bottom) of 338 longitudinal section layers across the entire placenta with a depth of 2670 µm. Scale bars are 500 µm. **b**) Whole-organ imaging (top) of cleared mouse tumor tissue stained with Emcn and α-SMA acquired on a light-sheet microscope. The representative section gallery views (bottom) of 295 longitudinal section layers across the entire tissue with a depth of 2563 µm. Scale bars are 300 µm. **c**) Whole-organ imaging (top) of cleared mouse salivary gland stained with KLK1 and DAPI. The representative section gallery views (bottom) of 522 longitudinal section layers across the entire salivary gland with a depth of 2538 µm. Scale bars are 500 µm.

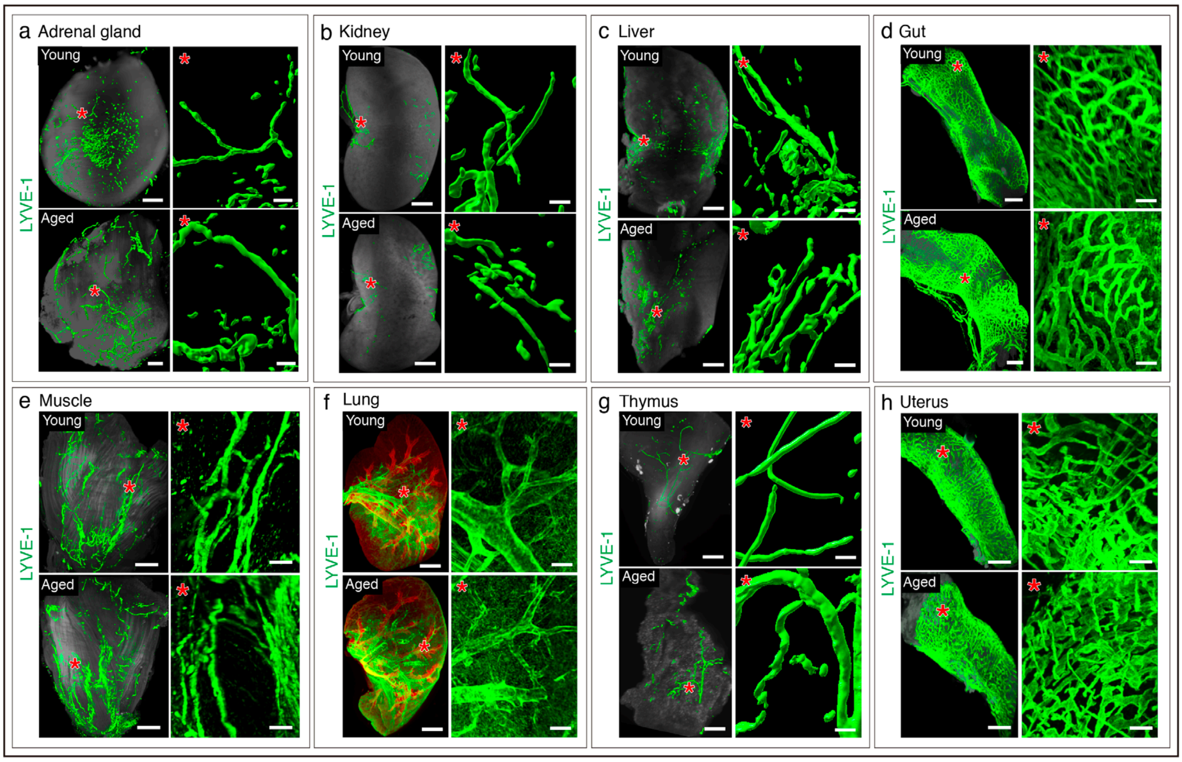

Figure S8. Lymphatic vessel imaging in young versus aged murine organs. a-h) Whole-organ imaging of cleared mouse adrenal gland, kidney, liver, gut, muscle, lung, thymus and uterus with LYVE-1 immunostaining. Asterisks indicate high magnification of specific areas in the different organs. Scale bars are 400 µm for whole-organ images and 50 µm for the high magnification insets.
