# Supplementary material for: Rapid 3D Immunolabeling and Light Sheet Microscopy for Quantitative Analysis of Intact Tissues
Source: Comput Struct Biotechnol J. 2026 May 21;35(1):0121. doi: 10.34133/csbj.0121 (PMC13191089; doi:10.34133/csbj.0121)
Supplement: Supplementary 1 — Figs. S1 to S8 Tables S1 to S4 Movies S1 to S5 [file csbj.0121.f1.zip › Table S1. Ab list.pdf]

Table S1. List of primary antibodies thoroughly tested with this method

ANTIBODY	ALTERNATIVE NAME	CLONE	SPECIES	COMPANY	CAT NO.	CELL TYPE
α-SMA	ACTA1	IA4	Mouse	Sigma-Aldrich	C6198	Artery EC; Myoepithelial cell
Endomucin	Endomucin-2	V.7C7	Rat	Santa Cruz	sc-65495	EC
Collagen IV	COL4A3	Poly	Rabbit	Chemicon	AB756P	Matrix; EC
HSPG2	PLC	A7L6	Rat	Millipore	MAB1948P	Matrix
Isolectin	Isolectin B4	IB4	Biotinylated Griffonia	Vector Lab	B-1205	EC
FSP1	Calvasculin	Poly	Rabbit	Millipore	07-2274	Fibroblast
Pan Cadherin	Cadherin-1	EPR1792Y	Rabbit	Abcam	ab195203	EC
CD146	MCAM	EPR3208	Rabbit	Abcam	ab196448	EC
SM22α	Transgelin	Poly	Rabbit	Abcam	ab14106	Vascular SMC
CD144	VE-cadherin	Poly	Goat	R&D Systems	AF1002	EC
CD102	ICAM-2	3C4(mIC2/4)	Rat	BD Pharmingen	553326	EC
CD34	CD34 Molecule	RAM34	Rat	eBioscience	14-034-82	HSC; EC
CD31	PECAM1	MEC13.3	Rat	BD Biosciences	553370	EC
CD31	PECAM1	Poly	Goat	R&D Systems	FAB3628G	EC
Endoglin	CD105	Poly	Goat	R&D Systems	AF1320	EC
Endoglin	CD105	Poly	Goat	R&D Systems	AF1097	EC
Podocalyxin	PCLP1	Poly	Goat	R&D Systems	AF1556	EC
Decorin	DCN; PG40	Poly	Goat	R&D Systems	AF1060	MC
FABP4	A-FABP	Poly	Goat	R&D Systems	A1443	Adipocytes
CD44	PGP-1	IM7	Rat	BioLegend	103016	Multiple cell types
ESM-1	Endocan	Poly	Goat	R&D Systems	AF1999	EC/Tip cell
ESAM	LP4791 Protein	340236	Rat	R&D Systems	MAB28271	EC
Ki67	MKI67	Poly	Rabbit	Abcam	ab15580	Mitotic chromosome
Vinculin	VCL	EPR20407	Rabbit	Abcam	ab219649	Matrix
VCAM-1	CD106	Poly	Goat	R&D Systems	AF643	EC
E-Cadherin	CD324	Poly	Goat	R&D Systems	AF748	EC

Smoothelin	SMTN	EPR20044	Rabbit	Abcam	ab219652	SMC
Perilipin	PLIN1	D1D8	Rabbit	Cell signaling	9349	Adipocytes
NG2	CSPG4	Poly	Rabbit	Millipore	AB5320	Pericytes
P-selectin	SELP; CD62P	Poly	Goat	R&D Systems	AF737	EC
Laminin	LAMA1	Poly	Rabbit	Sigma-Aldrich	L9393	Matrix
ICAM-1	CD54	Poly	Goat	R&D Systems	AF796	EC
ICAM-1	CD54	EP1442Y	Rabbit	Abcam	ab53013	EC
TER-119	Ly-76	Mono	Rat	BioLegend	116202	Erythroid cells
LYVE-1	Extracellular link domain containing 1; XLKD1	Poly	Rabbit	Abcam	ab14917	Lymphatic EC; Macrophage
PROX1	Prospero-related homeobox 1	Poly	Rabbit	Abcam	ab101851	Lymphatic EC
Caveolin-1	CAV1; MSTP085	Poly	Rabbit	Cell signaling	3238	EC
KLK1	Kallikrein 1	Poly	Sheep	R&D Systems	AF7928	Epithelial cells
