# Supplementary material for: Rapid 3D Immunolabeling and Light Sheet Microscopy for Quantitative Analysis of Intact Tissues
Source: Comput Struct Biotechnol J. 2026 May 21;35(1):0121. doi: 10.34133/csbj.0121 (PMC13191089; doi:10.34133/csbj.0121)
Supplement: Supplementary 1 — Figs. S1 to S8 Tables S1 to S4 Movies S1 to S5 [file csbj.0121.f1.zip › Table S2. Human sample.pdf]

Table S2. Details of healthy young and aged human endocrine gland tissue samples

Serial Number	Sample Name	Sample AMSBIO ID	Cataloge number	Age	Gender	Diagnosis	Ethnicity	Year of Procurement
1	Ovary	4765	AMS-33028	19	female	normal	Caucasian	09/11/2016
2	Ovary	5514	AMS-33028	18	female	normal	Caucasian	19/07/2015
3	Ovary	6379	AMS-33028	18	female	normal	Caucasian	03/12/2016
4	Ovary	7456	AMS-33028	19	female	normal	Caucasian	15/05/2017
5	Ovary	2108	AMS-33028	80	female	normal	Caucasian	30/07/2017
6	Ovary	1582	AMS-33028	79	female	normal	Caucasian	13/03/2017
7	Ovary	6715	AMS-33028	70	female	normal	Caucasian	12/01/2015
8	Ovary	4517	AMS-33028	72	female	normal	Caucasian	28/08/2016
9	Testis	149	AMS45022	18	male	normal	Caucasian	19/04/2016
10	Testis	152	AMS45022	19	male	normal	Caucasian	03/05/2017
11	Testis	154	AMS45022	18	male	normal	Caucasian	25/05/2017
12	Testis	156	AMS45022	20	male	normal	Caucasian	11/09/2018
13	Testis	29300	AMS45022	70	male	normal	Caucasian	22/09/2014
14	Testis	3509	AMS45022	71	male	normal	Caucasian	01/07/2014
15	Testis	3077	AMS45022	76	male	normal	Caucasian	27/08/2017
16	Testis	7267	AMS45022	75	male	normal	Caucasian	18/03/2017
17	Thyroid gland	0235	AMS-47028	18	female	normal	Caucasian	27/09/2015
18	Thyroid gland	3555	AMS-47028	19	female	normal	Caucasian	19/06/2015
19	Thyroid gland	5279	AMS-47028	19	female	normal	Caucasian	20/04/2015
20	Thyroid gland	6022	AMS-47028	20	female	normal	Caucasian	03/10/2016
21	Thyroid gland	4274	AMS-47028	71	female	normal	Caucasian	20/05/2016
22	Thyroid gland	3712	AMS-47028	73	male	normal	Caucasian	01/12/2015
23	Thyroid gland	2640	AMS-47028	70	female	normal	Caucasian	23/01/2016
24	Thyroid gland	4888	AMS-47028	72	female	normal	Caucasian	17/02/2017
