# Supplementary material for: Rapid 3D Immunolabeling and Light Sheet Microscopy for Quantitative Analysis of Intact Tissues
Source: Comput Struct Biotechnol J. 2026 May 21;35(1):0121. doi: 10.34133/csbj.0121 (PMC13191089; doi:10.34133/csbj.0121)
Supplement: Supplementary 1 — Figs. S1 to S8 Tables S1 to S4 Movies S1 to S5 [file csbj.0121.f1.zip › Table S3. Troubleshooting table.pdf]

Table S3. Troubleshooting table

Problems	Possible reason	Solution
Weak antibody signal	Antibody concentration is inadequate to stain the whole organ	Optimize the antibody concentration
Noisy and non-transparent fluorescence signal	The dehydration may be limited. It also affects tissue clearing	Ensure the dehydration is enough to get transparent signal as per protocol
Antibody precipitations on the organ	Inadequate washing	Ensure adequate wash is carried out to overcome this issue
Appearance of nonspecific fluorescence signal	Precipitates and aggregates in the secondary antibodies	Avoid freeze-thaw cycles and the antibody should be spun down and mixed gently before use
High background along with antibody signal	Too much pigmentation and Hematoma on samples; Inadequate washing steps	Bleaching required to remove pigmentation and reduce hematoma signal; Wash more times
Fade antibody staining	Dehydration of chemicals	Optimised dehydration as suggested on the protocol
Tissue clearing and preservation of fluorescence signal	Slow tissue clearing and loss of fluorescence signal within few days	PEG increases the hydration capacity of the clearing medium and preserve longer fluorescence signal
Sometimes bubbles build up inside the whole organ	Hole in the organ and last hydration step	Fill the empty space in the organ with clearing solution by needle without damaging the tissues
Unstained or dark regions toward the centre to the organ/tissue	Collagenase digestion was either insufficient or unsuccessful	Collagenase solution should be freshly prepared; collagenase should be incubated at 37°C with constant shaking
