# Supplementary material for: Rapid 3D Immunolabeling and Light Sheet Microscopy for Quantitative Analysis of Intact Tissues
Source: Comput Struct Biotechnol J. 2026 May 21;35(1):0121. doi: 10.34133/csbj.0121 (PMC13191089; doi:10.34133/csbj.0121)
Supplement: Supplementary 1 — Figs. S1 to S8 Tables S1 to S4 Movies S1 to S5 [file csbj.0121.f1.zip › Table S4.pdf]

Table S4. Estimated voxel size and minimum detectable structure size

Imaging mode	Representative sample	Structure/channel	Estimated voxel size, µm³	Estimated minimum detectable structure size	Intended quantitative use
Light-sheet, whole-organ overview	Lung	Blood vessels	~3.25 × 3.25 × 12.1	~15–20 µm tubular structures	Whole-organ vascular architecture and vessel density
Light-sheet, whole-organ overview	Gut / uterus	Lymphatic vessels	~3.25 × 3.25 × 10–12	~15–20 µm tubular structures	Whole-organ vascular architecture and vessel density
Light-sheet, ROI/high-zoom acquisition	Thymus / testis	α-SMA^+ cells or $CD8a^+$ cells	~1.6 × 1.6 × 5–8	~8–10 µm cellular structures	Region-specific cell or small-structure visualization
Confocal tile scan	Endocrine tissues	$DAPI^+$ nuclei, $Prox1^+$ cells	~0.8–1.3 × 0.8–1.3 × 2–5	~5–7 µm nuclei/cells	Cell-level validation and counting
